# Effect of robotic assistance on upper-limb sensorimotor recovery: a systematic review and meta-analysis

**DOI:** 10.3389/fnbot.2026.1860365

**Published:** 2026-10-20

**Authors:** Ritwika Choudhuri, John M. Solomon, Aravind Nehrujee, S. Sujatha, Sivakumar Balasubramanian

**Affiliations:** 1 Department of Bioengineering, Christian Medical College Vellore, Vellore, India; 2 Department of Mechanical Engineering, Indian Institute of Technology Madras, Chennai, India; 3 Department of Physiotherapy, Manipal College of Health Professions, Manipal Academy of Higher Education, Manipal, India; 4 Shirley Ryan AbilityLabs, Chicago, IL, United States; 5 Department of Physical Medicine and Rehabilitation, Northwestern University, Chicago, IL, United States; 6 School of Health and Rehabilitation Sciences, The University of Queensland, Brisbane, QLD, Australia

**Keywords:** neurorehabilitation, rehabilitation robots, robotic assistance, sensorimotor recovery, stroke, upper-limb

## Abstract

**Introduction:**

Robot-assisted upper-limb neurorehabilitation enables several beneficial therapy components, including repetitive task-specific practice, high training intensity, real-time feedback, gamification, and continuous physical interaction with the patient’s limb. However, conventional comparisons of robot-assisted and therapist-delivered rehabilitation cannot determine whether the physical assistance provided by the robot contributes independently to recovery. This systematic review and meta-analysis, therefore, focused specifically on randomized controlled trials (RCTs) of upper-limb rehabilitation in which experimental and comparator interventions were designed to be similar except for the presence, or level, or mode of robotic assistance.

**Methods:**

PubMed, Scopus, and Google Scholar were searched through November 2025 for eligible studies. Study screening and filtration were performed using Covidence software. Thirteen studies met the eligibility criteria for qualitative synthesis, and nine studies involving 272 stroke survivors provided Fugl-Meyer Assessment (FMA) data for quantitative synthesis. The standardized mean difference (SMD) between the control and the experimental group for change in pre-to-post intervention FMA score was calculated to perform meta-analysis. Heterogeneity computation, sensitivity analysis, risk of bias, publication bias, and certainty of evidence evaluation were also performed.

**Results:**

The main meta-analysis pooled effect (SMD = 0.425) favored the experimental robot-assisted condition across disease stages, assistance levels, and targeted limb segments. However, this overall effect was imprecise (95% CI: [−0.007, 0.858]) and showed substantial heterogeneity (62.62%). In studies restricted to chronic stroke, the pooled effect was larger (SMD = 0.53), although imprecision [0.067, 0.992] and heterogeneity (60.72%) remained similar. Estimates for shoulder-elbow and hand-focused interventions were small and imprecise too. Sensitivity analyses showed that the direction of the pooled effect was stable across various considerations. The certainty of evidence was judged to be low, primarily because of risk of bias concerns (mainly lack of double-blinding), inconsistency, and imprecision. Exploratory meta-regression did not identify clear study-level moderators of between-study heterogeneity. In addition, no significant publication bias was identified.

**Discussion:**

Although this study provides preliminary evidence suggesting a positive independent influence of the robotic assistance on upper-limb recovery, the limited number of studies with their small sample sizes and substantial heterogeneity preclude a firm conclusion. Larger, adequately powered RCTs that isolate robotic physical assistance while standardizing other intervention components are needed to establish its independent causal contribution to upper-limb sensorimotor recovery.

## Introduction

Robot-assisted upper-limb neurorehabilitation has been an active area of research for more than two decades (Volpe et al., 2000), leading to the development of a wide range of end-effector and exoskeleton systems (Maciejasz et al., 2014; Mehrholz et al., 2018) for shoulder, elbow, wrist, hand, and whole-arm training. These systems can deliver repetitive and intensive training while physically interacting with the patient’s limb to assist, guide, resist, or otherwise modify movement for sensorimotor recovery (Marchal-Crespo and Reinkensmeyer, 2009; Yue et al., 2017; Lo et al., 2010; Reinkensmeyer et al., 2001; Krebs et al., 2003; Hogan et al., 2006; Warraich and Kleim, 2010). The overall effectiveness of robot-assisted upper-limb rehabilitation has been examined extensively, and systematic reviews (Bertani et al., 2017; Lee et al., 2023; De Iaco et al., 2024; Park et al., 2025) indicate that robotic rehabilitation can improve upper-limb motor outcomes, although average effects are generally modest.

Given that physical assistance or guidance is a core component of robot-assisted therapy, a fundamental question to ask is whether the assistance provided by rehabilitation robots contributes independently to sensorimotor recovery. Understanding this independent contribution is important both clinically, for designing appropriate robot-assisted rehabilitation protocols, and technically, for guiding the design and control of rehabilitation robots. However, this question is difficult to address using randomized controlled trials (RCTs) comparing robot-assisted therapy with conventional therapy because the two interventions differ in several respects beyond robotic assistance, including the movements trained, treatment intensity, dose, feedback, and training environment (Mehrholz et al., 2018; Reinkensmeyer et al., 2001). Therefore, any observed difference in recovery cannot necessarily be attributed specifically to the physical assistance provided by the robot.

To isolate the contribution of robotic assistance more directly, comparisons are needed between interventions in which the experimental and control groups perform comparable tasks under similar training conditions and differ principally in the presence, level, or mode of robotic assistance. Examples include assisted versus unassisted training, high versus low assistance, fixed guidance versus assist-as-needed control, and movement-intention-contingent versus non-contingent assistance. To the best of our knowledge, no previous review has specifically aimed to determine the effect of robotic assistance on upper-limb sensorimotor recovery by synthesizing RCTs designed to isolate the assistance component of robot-assisted rehabilitation. Even recent reviews that moved beyond evaluating the overall efficacy of robot-assisted rehabilitation to examining factors that may influence treatment outcomes, including robotic device characteristics, anatomical target, training dose, post-stroke stage, and training modality, retained conventional therapy as the comparator (Boardsworth et al., 2025; Alvaro et al., 2026; Wang et al., 2025) and did not specifically isolate the independent contribution of robotic physical assistance. Therefore, this systematic review primarily aimed to determine the contribution of robotic physical assistance by collating RCTs of upper-limb rehabilitation in which the experimental and control groups received otherwise comparable training conditions but differed principally in the presence, level, or mode of robotic assistance. Secondary objectives were to examine whether the estimated effect differed according to post-stroke stage or the anatomical target of training.

## Methods

This systematic review and meta-analysis was conducted and reported in accordance with the Preferred Reporting Items for Systematic Reviews and Meta-Analyses (PRISMA) 2020 framework (Page et al., 2020), which includes 27 items for transparent reporting of systematic reviews and an associated flow diagram. The present study protocol was not prospectively registered in the International Prospective Register of Systematic Reviews (PROSPERO) or an equivalent registry. The decision not to register reflected the exploratory, mechanism-focused framing of the review question (isolating the independent effect of robotic assistance), which differs from a conventional intervention versus comparator systematic review and was defined only after initial scoping of the eligible literature. We acknowledge this as a limitation of methodological transparency and recommend that future updates of this review, or related reviews on this topic, be prospectively registered.

### Search strategy

The study extraction was conducted using three electronic bibliographic databases: PubMed, Scopus, and Google Scholar up to November 2025. Various keyword combinations synonymous with the primary Medical Subject Heading (MeSH) terms—robot, rehabilitation, and upper-limb—were used to define the search strategy. In each database, the search was conducted using a Boolean logic rule for the keywords such as “robot-assisted OR robotic OR robot-based OR end-effector OR exoskeleton” AND “rehabilitation OR neurorehabilitation OR therapy OR training” AND “upper limb OR upper extremity OR arm OR forearm OR shoulder OR elbow OR hand OR finger” NOT “review” etc. (detailed keywords and search formulas are provided in the Supplementary material, Section I). These terms were searched for in the titles, abstracts, and keywords of the literature. References identified through database searches and additional citation searches were imported into Covidence (2014) software for duplicate removal and screening.

### Eligibility criteria

The studies were screened by reviewing their titles, abstracts, and, where necessary, the full texts. Studies were excluded if participants were younger than 18 years or had upper-limb impairment of primarily musculoskeletal origin; or if the intervention targeted the lower limb, gait, balance, or whole-body rehabilitation; or if the experimental and comparator conditions differed systematically in therapeutic components other than the presence, level, or mode of robotic assistance. Accordingly, studies comparing different treatment doses or intensities, feedback strategies, training contexts, or robotic technologies were excluded.

Additionally, studies were excluded where subjective factors such as active participation and motivation of the participants differed between the groups. When these differences were not explicitly reported by the authors, an “implicit difference” was inferred as a between-group difference in the intervention that could independently influence the study outcomes, thereby preventing isolation of the effect of robotic assistance. Such differences were identified from the reported intervention protocols of the respective study. For example, studies (Hu et al., 2009; Murakami et al., 2023) comparing active versus passive assistance were excluded because only the active condition required volitional movements from the participant, thus inducing a major difference between the experimental and control groups. Assistive versus resistive training (Fasoli et al., 2003; Stein et al., 2004; Jeon et al., 2024) was excluded because interaction with a resistive controller requires additional voluntary effort to overcome the applied resistance compared to the assistive condition. Likewise, error-augmenting versus passive or error-reducing training (Givon-Mayo et al., 2014; Bouchard et al., 2017) was excluded because the two compared paradigms imposed different task demands and levels of voluntary engagement; error plays a vital role in motor and skill learning; thus, manipulation of error can confound the effects of robotic assistance. These differences could independently influence active participation, motivation, and learning between the groups and therefore confound the isolated effect of robotic assistance. Assessment of potential implicit between-group differences was performed independently by R.C. and S.B. during full-text screening using the predefined criterion that any between-group difference capable of independently affecting voluntary engagement, task demands, motor learning, or treatment exposure precluded isolation of robotic assistance. Disagreements or borderline cases were discussed between the two reviewers and resolved by consensus.

Non-randomized studies, case reports, reviews, and abstracts-only literature were also excluded. Detailed selection criteria are outlined in Table 1 using the Population–Intervention–Comparator–Outcome (PICO) framework.

**Table 1:** List of inclusion and exclusion criteria for determining the eligibility of randomized controlled trials on upper-limb robotic therapy to be shortlisted for the systematic review.

Domain	Inclusion criteria	Exclusion criteria
Population	People having upper-limb impairment due to neurological disorders such as stroke, spinal cord injury, multiple sclerosis, and traumatic brain injury Age of participant ≥ 18 years	People having upper-limb impairment due to musculoskeletal disorders Age of participant < 18 years
Intervention	Robot-assisted therapy for upper-limb neurorehabilitation (active assistance, assist-as-needed, high assistance, assistance contingent on movement intention, progressive arm weight support, deficit force field, negative viscous force) Both experimental and control groups in a study were exposed to the same robotic device for therapy Intensity and dosage of the therapy were the same between the groups Nature and schedule of the feedback were the same between the groups	Robot-assisted therapy for hip, knee, lower-limb, balance, and gait Error-augmenting robotic therapy Subjective factors such as active participation and motivation of the participants differed between the groups (either reported by the authors of the study or inferred by the reviewers)
Comparator	Unassisted, predefined amount of assistance, low assistance, virtual assistance, assistance non-contingent on movement intention, no or full arm weight support, and null force field	End-effector versus exoskeleton, proximal versus distal robots, or two same purpose robots with different features or of different brand Conventional therapy versus robot-assisted therapy Low versus high-intensity robotic training
Outcomes	Measure of impairment Measure of activity and/or performance	
Study	Randomized controlled trials (RCTs) Published in peer-reviewed journals Pre-print of an original research article (gray literature)	Case study or abstract only Review study
Language	English	

### Study selection

Two authors (R.C and S.B) independently screened the references and resolved the conflicts through discussion, resulting in 23 studies being shortlisted. Among these, two studies on active versus complete passive assistance (Hu et al., 2009; Murakami et al., 2023) (level of voluntary engagement differed between the groups), three studies on assistive versus resistive robotic therapy (Fasoli et al., 2003; Stein et al., 2004; Jeon et al., 2024) (resistive group required additional voluntary effort to overcome the applied resistance), and two studies that involved error-augmenting training (Givon-Mayo et al., 2014; Bouchard et al., 2017) (compared paradigms imposed different task demands and levels of voluntary engagement between the groups) were further excluded because the active participation of the participants between the control and experimental groups appeared to be implicitly different in these studies. Moreover, one study on high versus low robotic assistance (Takebayashi et al., 2022), which apparently qualified the eligibility criteria, was further eliminated, as it was a retrospective analysis of data from an RCT (Takahashi et al., 2016), in which the control group received self-guided therapy, and the assignment of the level of robotic assistance to the individuals in the experimental group was not randomized. One more study (Wright et al., 2015) that satisfied the eligibility criteria was also excluded because it reported preliminary data from another study (Wright et al., 2018) that has already been included in this review. An additional relevant study (Pila et al., 2021) was removed because it appeared to be part of a larger trial for which no detail or record could be found online. Hence, due to the lack of clarity on the study’s randomization and blinding procedures, it was not included in the core synthesis of this review. However, the main meta-analysis was repeated, including the data from this study (Pila et al., 2021), and the corresponding result is reported in Section IV of the Supplementary material. In total, 13 studies were finally included for this systematic review, and data from nine studies, all involving stroke survivors, were used for the meta-analysis. A flow diagram of the study selection process is shown in Figure 1.

**Figure 1:**
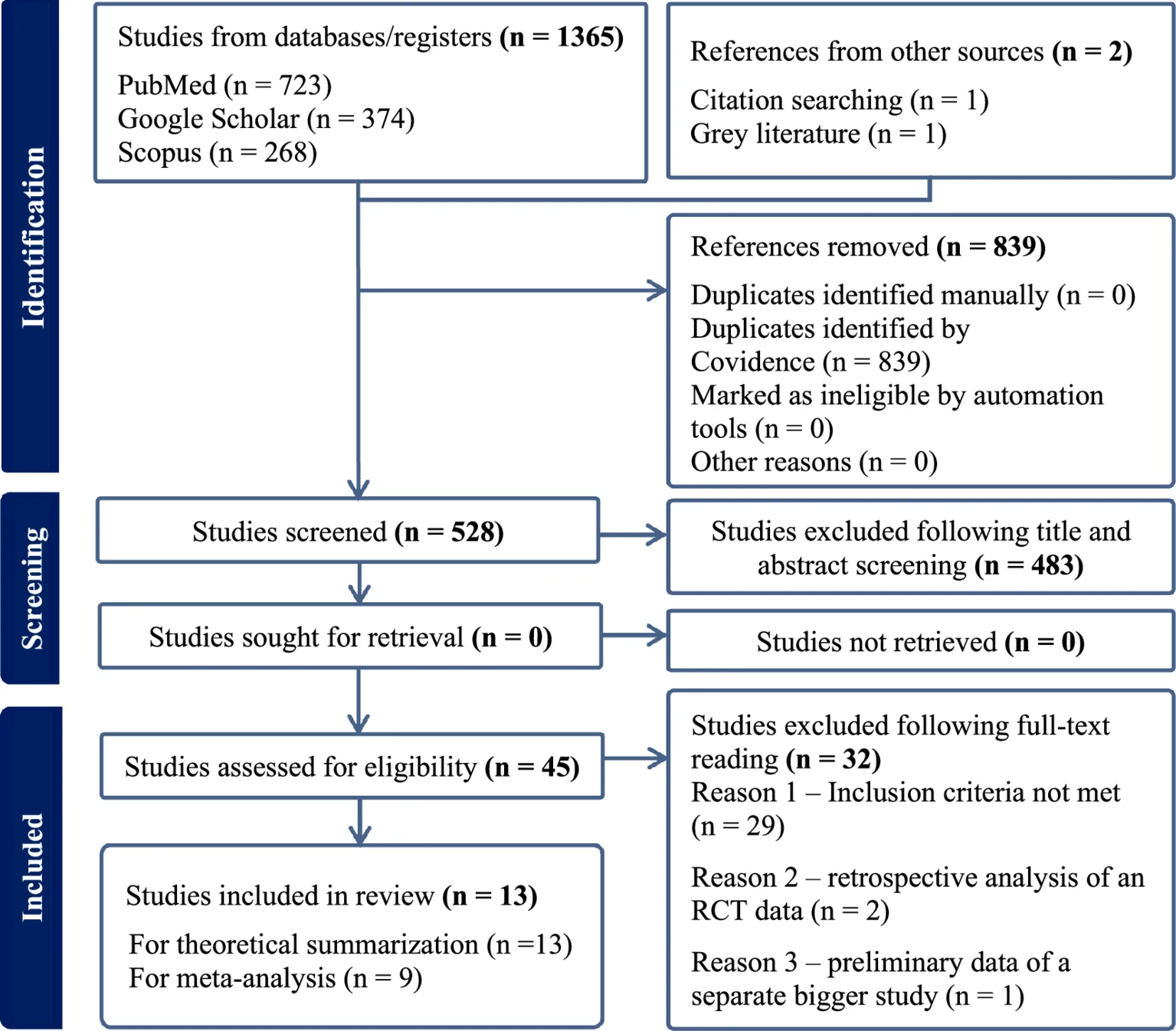
PRISMA flow diagram depicting the steps of the study selection process.

### Data extraction

Data extraction was performed by author R.C, who recorded the following information for each included study into a standardized spreadsheet: number of subjects, time from stroke onset, robot employed, experimental protocol, therapy intensity, outcome measures, and change in pre-to-post intervention FMA score (impairment measure). A senior author (S.B) independently verified the extracted data against the source publications. Discrepancies between the two authors were resolved by discussion and, where necessary, consultation with a third author until consensus was reached; no discrepancies required external adjudication. Details of the extracted information are presented in Tables 2, 3 of this document along with Section II and Section III of the Supplementary material.

**Table 2:** Relevant information extracted from the studies included in this systematic review.

Studies	Disease stage and severity	Experimental group	Control group	Device employed and therapy duration	Tasks performed during robot-assisted therapy	Outcome measures	Usual therapy?
Takahashi et al. (2008)	Chronic stroke Severity—moderate	Active assistance for all sessions (*N* = 7)	Half of the sessions were unassisted but connected to the device; the other half of the sessions were active assistance (*N* = 6)	Hand-wrist assistive rehabilitation device (“HWARD”) 15 sessions (1.5 h/session) over 3 weeks	Hand in grasp and release movement (wrist and finger flexion-extension)	ARAT, FMA, BBT, ROM, MAS, NHPT, EMG, fMRI	Not mentioned
Ellis et al. (2009)	Chronic stroke (greater than 6 months after stroke) Severity—moderate to severe	Robotic training (no assistance) with progressive arm weight support (*N* = 7)	Robotic training (no assistance) with full arm weight support (*N* = 7)	Haptic MASTER 3 sessions, in a week over 8 weeks	Reaching movement to targets in five directions (six torque conditions) involving shoulder abduction-adduction, shoulder flexion, and elbow flexion-extension	Work area and isometric strength	Not mentioned
Huang and Patton (2013)	Chronic stroke (greater than 1 year to less than 9 years post-stroke) Severity—severe	Two equal subgroups (cross over design)—null force, negative viscous force, and negative viscous force with inertia (*N* = 20)	Null force field (*N* = 10)	Planar force—feedback device 3 sessions, each of 2 h 15 min	Any movement of own choice using a variety of directions, speed and positions within a rectangular workspace (exploratory movement)	Movement quality and performance accuracy in terms of trajectory path, speed, and acceleration	Not mentioned
Ramos-Murguialday et al. (2013)	Chronic stroke (greater than 6 months post-stroke) Severity—severe	Active assistance contingent on movement intension (*N* = 16)	Active assistance non-contingent on movement intension (*N* = 14)	Hand orthosis with BCI (EEG based) 20 sessions (5 sessions/week); total 4 weeks	Reach and grasp tasks involving upper arm (shoulder-elbow flexion and extension) movement to reach forward and finger flexion-extension (hand) to grasp. Considered whole arm training	FAM, MAL, GAS, EMG, fMRI	Yes
Susanto et al. (2015)	Chronic stroke (6 to 24 months post-stroke) Severity—moderate	Robot assisted (*N* = 9)	Unassisted (*N* = 10)	A custom-designed hand exoskeleton robot; 5 individual digits powered by 5 linear actuators 3–5 times, 1 h session per week; 20 sessions in 5 weeks	Finger flexion and extension (hand grasping-opening, three-finger and two-finger pinching-opening)	ARAT, WFMT, FMA, and finger individuation index	Not clear
Frolov et al. (2017)	Subacute stroke (1–6 months post-stroke) and chronic stroke (greater than 6 months post-stroke) Severity—severe	Assistance dependent on motor imagery-based EEG activity (*N* = 55)	Assistance non-dependent on motor imagery-based EEG activity; sham BCI (*N* = 19)	A custom-designed BCI-controlled hand exoskeleton 10 sessions; each lasting up to 40 min; over 2–3 weeks	Three mental tasks—motor relaxation, motor imagery of paretic hand opening, and motor imagery of non-paretic hand opening. BCI active—the task-specific imagination brain activity drove the exoskeleton attached to the paretic hand BCI passive-random actuation of the exoskeleton	ARAT and FMA	Yes
Frullo et al. (2017)	Chronic incomplete spinal cord injury (SCI) Severity—moderate to severe	Assist as assisted (*N* = 7)	Self-triggered (partially passive assistance) (*N* = 7)	MAHI EXO-II (upper extremity exoskeleton) 10 sessions (90 min each), 1–3 sessions per week, all sessions over 3–4 weeks	Point to point movement from a center target to peripheral targets involving elbow flexion-extension and wrist radial-ulnar deviation	ARAT, MAS, grip-pinch strength, GRASSP and movement kinematics	Not mentioned
Rowe et al. (2017)	Chronic stroke (at least 6 months post-stroke) Severity—moderate	High assistance; caused average 82% success rate (*N* = 15)	Low assistance; caused average 55% success rate (*N* = 15)	FINGER exoskeleton 1 h/session; 3 times in a week for 3 weeks; total 9 sessions	Index and middle finger flexion-extension	BBT, FMA, ARAT, MAS, MAL, IMI	Not mentioned
Wright et al. (2018)	Chronic stroke (greater than 8 months post-stroke) Severity— moderate to severe	Assist-as-needed; deficit force field (*N* = 11)	Unassisted; null force field (*N* = 11)	A custom-designed planar robotic manipulandum 3 days/week (2–7 sessions) for 2 weeks; each session of 33–35 min including 1–3 min rest	Planar shoulder-elbow movement (exploratory movement task)	FMA, ARAT, MAS, CMAS-S, and elbow ROM	Not mentioned
Cho and Song (2019)	Chronic stroke (at least 6 months post-stroke) Severity—mild	Assist-as-needed force (*N* = 19)	Guidance force; constant fixed assistance but not completely passive (*N* = 19)	Whole arm manipulandum (WAM) 40 min per day, 3 days in a week for 6 weeks; total 18 sessions	Reaching movement toward targets in 3D space in 6 directions involving shoulder and elbow movements	FMA, ARAT, BBT, and movement velocity	Not mentioned
Solaro et al. (2020)	Multiple Sclerosis	Haptic—robot assisted (*N* = 19)	Sensorimotor-visual control- no force applied by the robot (*N* = 17)	Planar robotic manipulandum 40 min per session, 2 sessions per week for 6 weeks	Fast and accurate reaching movements in 6 different directions	9HPT and ARAT	Not mentioned
Park et al. (2020)	Subacute and Chronic stroke (greater than 3 months post-stroke) Severity—moderate	Active robotic assistance with gravity compensation (*N* = 10)	Unassisted; mechanical support with gravity compensation by the robot; no actuation (*N* = 9)	Armeo Power (for active assistance group) and Armeo Spring (for passive group) 30 min session/day, 5 days in a week for 4 weeks (total 20 sessions)	Virtual reality games involving shoulder, elbow and forearm movements	WFMT, FMA, SIS, and movement kinematics	Yes
Farrens et al. (2025a)	Chronic stroke (greater than 6 months post-stroke) Severity—mild to moderate	Active robotic assistance (self-initiated movement with external visual feedback) *N* = 15	Non-robotic assistance (complete self-generated movement with external visual feedback; virtual assistance through amplifying display movements and by adjusting gaming speed and accuracy requirements) *N* = 15	FINGER robot 3 weeks hand movement training (9 sessions, each 2 h, ~1,050 movements/ session at 80% success rate)	Game-based training involving flexion-extension of index and middle fingers and thumb circumduction	BBT, FMA, NHPT, MAS, MAL, VAS-pain, hand capacity and task error, and EEG	Not mentioned

BCI, Brain–Computer Interface; FMA, Fugl-Meyer Assessment; CMSA, Chedoke-McMaster Stroke Assessment; MSS, Motor Status Scale; BBT, Box and Block Test; NHPT, Nine Hole and Peg Test; WFMT, Wolf Motor Function Test; FIM, Functional Independence Measure; ARAT, Action Research Arm Test; MAL, Motor Activity Log; MAS, Modified Ashworth Scale; FAS, Functional Assessment Scale; GRASSP, Graded Redefined Assessment of Strength; Sensibility; and Prehension Test; SIS, Stroke Impairment Scale; GAS, Goal Assessment Scale; ROM, range of motion; VAS, Visual Analog Scale; EMG, Electromyography; fMRI, Functional Magnetic Resonance Imaging; EEG, Electroencephalography.

**Table 3:** Relevant data extracted from studies for the nine studies included in the meta-analysis.

Studies	Disease stage	No. of subjects	Outcome measure	Pre-to-Post (immediate) intervention FMA score change (μ ± SD)	SMD with 95% confidence interval	SE of SMD
Takahashi et al. (2008)	Chronic	EG: 7 CG: 6	FMA	EG: 9.1 ± 2.1 CG: 5.8 ± 1.6	1.625 (0.302, 2.947)	0.675
Ramos-Murguialday et al. (2013)	Chronic	EG: 16 CG: 14	Modified FMA	EG: 3.4 ± 0.563 CG: 0.35 ± 2.24	1.877 (0.997, 2.756)	0.448
Susanto et al. (2015)	Chronic	EG: 9 CG: 10	FMA	EG: 5.11 ± 6.55 CG: 5.7 ± 4.35	−0.103 (−1.003, 0.798)	0.459
Frolov et al. (2017)	Subacute + Chronic	EG: 55 CG: 19	FMA	EG: 4.67 ± 17.83 CG: 2.66 ± 22.53	0.104 (−0.417, 0.625)	0.266
	Chronic	EG: 30 CG: 10	FMA	EG: 7.17 ± 18.21 CG: 4.0 ± 24.33	0.156 (−0.559, 0.873)	0.365
Rowe et al. (2017)	Chronic	EG: 15 CG: 15	FMA	EG: 2.7 ± 2.6 CG: 2.2 ± 3.5	0.157 (−0.559, 0.874)	0.366
Wright et al. (2018)	Chronic	EG: 10 CG: 9	FMA	EG: 1.1 ± 1.54 CG: 1 ± 1.69	0.059 (−0.841, 0.96)	0.459
Cho and Song (2019)	Chronic	EG: 19 CG: 19	FMA	EG: 4.0 ± 2.58 CG: 2.0 ± 2.60	0.755 (0.095, 1.416)	0.337
Park et al. (2020)	Subacute + Chronic	EG: 10 CG: 9	FMA	EG: 2.58 ± 8.6 CG: 5.4 ± 7.48 (data extracted from graph using WebPlotDigitizer)	−0.333 (−1.241, 0.575)	0.463
Farrens et al. (2025a)	Chronic	EG: 15 CG: 15	FMA	EG: 3.83 ± 8.77 CG: 2.33 ± 5.34	0.201 (−0.516, 0.918)	0.366

EG, experimental group; CG, control group; FMA, Fugl-Meyer Assessment of the upper extremity; μ, mean; SD, standard deviation; SMD, standardized mean difference; CI, confidence interval; SE, standard error. The total score for FMA is 66, and for Modified FMA is 54 [the same outcome measure across studies, but a different scale was used in one study; therefore, SMD was considered instead of mean difference (MD)].

### Risk of bias and certainty of evidence evaluation

Risk of bias analysis of all 13 studies was performed manually for the signaling questions across five domains [as defined in the Cochrane risk of bias 2 framework (Higgins et al., 2024)]: bias arising from the randomization process, bias due to deviations from intended interventions, bias due to missing outcome data, bias in outcome measurement, and bias in selection of the reported result. The decision was solely made based on the individual judgment of author R.C. upon reading each study’s literature. The associated document link and the references are provided in Section III of the Supplementary material. The “traffic light” plot for visualizing the ROB report was generated using the open-source software: “robvis” (McGuinness and Higgins, 2021) and has been reported in the results section.

In addition, the overall certainty of evidence for the primary outcome (FMA change score) using the Grading of Recommendations, Assessment, Development, and Evaluations (GRADE) framework (Schünemann et al., 2019) was also evaluated considering risk of bias, inconsistency (heterogeneity), indirectness, imprecision, and publication bias.

### Statistical analysis

For meta-analyses, upper-limb impairment was selected as the primary outcome of interest, typically quantified using the Fugl-Meyer Assessment (FMA) score. FMA scores were pooled from 9 of the 13 included studies based on data availability. Among these nine studies, eight of them reported numerical outcome values directly, whereas for one study (Park et al., 2020), the data were extracted from a graphical representation of FMA scores using WebPlotDigitizer (Rohatgi, 2011). The mean and standard error of the pre-to-post-intervention change in FMA scores for both the experimental and control groups from figure 4c of the respective study (Park et al., 2020) were digitized in the mentioned software. To verify extraction accuracy, the plot was digitized independently twice by the same author (R.C), and the two sets of coordinate values were compared; extracted values differing by more than 2% were re-digitized. The final extracted values were cross-checked by another author (S.B).

The change in FMA scores (pre- to-post intervention) representing the intervention effect was mined for both the experimental and control groups as mean ± standard deviation (Table 3). Wherever the mean and standard deviation (SD) were not reported directly, the median instead of the mean and standard error or confidence interval or interquartile range instead of SD was provided, and the corresponding mean and SD were estimated using the respective conversion formulas described in Section III of the Supplementary material. Similarly, when pre- and post-intervention FMA scores were reported separately rather than as pre-to-post change scores, the mean change in FMA score and its corresponding SD were calculated using the formulas provided in Section III of the Supplementary material. The standardized mean difference (SMD—the effect size) of the primary outcome between the intervention outcomes of experimental and control groups was computed for the individual study.

Because all the included studies except one had small sample sizes (< 50), Hedges’ *g* SMDs with their 95% confidence intervals were calculated and pooled across the studies. Hedges’ *g* SMD includes pooled standard deviation to minimize the between-person variation across groups and a correction factor to reduce the small-sample upward bias (Lin and Aloe, 2021). Nevertheless, this correction does not completely eliminate the risk of effect-size inflation due to very small sample size. A few included trials had fewer than 15 participants per group (Table 3); therefore, effect-size estimates from these studies (e.g., Takahashi et al., 2008; Ramos-Murguialday et al., 2013) should be interpreted cautiously because they are particularly susceptible to sampling variability and potential overestimation of the true effect size despite the correction for the small-sample bias.

Based on Cohen’s criteria (Cohen, 1988), summary effect sizes were interpreted as small (0.2–0.49), moderate (0.50–0.79), or large (≥ 0.80). Both fixed-effect and random-effect models were used along with heterogeneity analysis (Deeks et al., 2024). Heterogeneity was quantified using the estimated between-study variance (τ^2^) and percentage of variability attributed to heterogeneity (I^2^). When the I^2^ > 50% (moderate level), the summary effect obtained by applying the random-effects model was considered, as it accounts for between-study variance, providing a more reliable summary effect size. The statistical significance was set at α = 0.05.

Meta-analysis was performed on the pooled data of all 9 studies to evaluate the overall intervention effect (i.e., effect of robotic assistance) irrespective of disease stage, the presence or absence of robotic assistance, the amount of assistance provided, and the targeted limb segment. However, subgroup analyses were performed on separate subsets of studies to determine the specific effect of robotic rehabilitation on chronic stroke survivors and to compare the intervention effect for therapy targeting shoulder-elbow versus therapy targeting hand (wrist or fingers). In addition, sensitivity analyses were performed by repeating the primary meta-analysis using different assumed pre-to-post measurement correlation coefficients (r = 0.5, 0.7, and 0.9) for the extracted data and by including data from a study (Pila et al., 2021) that had been excluded from the primary synthesis because of uncertainty regarding trial reporting and randomization.

Analysis of publication bias was also performed using funnel plot asymmetry and Egger’s regression analysis for the nine studies that were involved in the core meta-analysis; the corresponding details are mentioned in Section III (B) of the Supplementary material.

An exploratory random-effect meta-regression analysis was performed to investigate whether study-level characteristics could explain part of the between-study heterogeneity. Candidate moderators included chronic-only versus mixed-stage (chronic plus subacute) samples, anatomical target of training (hand, shoulder-elbow, and whole arm), disease severity (severe, moderate, mild, and mixed), and treatment dose. Given the limited number of studies, meta-regression analysis was restricted to univariable models to avoid overfitting and unstable estimates from simultaneous modeling of multiple moderators. Meta-regression analysis with fewer than ten studies is generally considered underpowered and is susceptible to spurious findings (Deeks et al., 2024); these analyses were therefore treated as hypothesis-generating rather than confirmatory. Details of this analysis are given in Section III (C) of the Supplementary material.

All the analyses were performed using Python. The code link and the used formulas along with their associated details are provided in Section III of the Supplementary material.

## Results

The database searches identified 1,365 records: 723 from PubMed, 374 from Google Scholar, and 268 from Scopus. Two additional records were identified through a citation search in literature and a gray literature (preprints) search. After removal of 839 duplicates, 528 records underwent title and abstract screening. Forty-five full-text reports were assessed for eligibility, and 13 randomized controlled trials were retained for qualitative synthesis. Nine trials provided sufficient FMA data for the primary meta-analysis.

### Qualitative synthesis

Thirteen controlled studies (sample sizes 13 to 74; total *N* = 369) examined robot-assisted upper-limb rehabilitation: 11 in stroke and 2 in other neurological disorders (spinal cord injury and multiple sclerosis). Nine of the stroke studies included participants in the chronic phase; two combined participants from both subacute and chronic phases. The therapy dose ranged from 4.5 to 28 h (3 to 30 sessions over 2 to 8 weeks). The combined findings demonstrated a reduction in post-therapy impairment in both control and experimental groups. However, a few studies (31% of total studies) have shown a significantly greater improvement in the experimental group, favoring assist-as-needed over constant assistance (Cho and Song, 2019), assistance contingent on movement intention over random assistance (Ramos-Murguialday et al., 2013), complete active assistance over a combination of active and no assistance (Takahashi et al., 2008), and physical assistance over virtual assistance (Farrens et al., 2025a).

#### Narrative summary

Active robotic assistance throughout hand grasp-release training produced greater functional and impairment recovery than an equal combination of assisted and unassisted training in moderately affected chronic stroke survivors, with significant within- and between-group effects (Takahashi et al., 2008). Robot-assisted shoulder-elbow reaching was also superior to unassisted therapy in multiple sclerosis survivors (Solaro et al., 2020). In contrast, active-assisted versus unassisted robotic hand training showed no significant group difference (Susanto et al., 2015).

Similarly, active robotic versus non-robotic assistance with gravity compensation showed no significant difference after shoulder-elbow training in subacute and chronic stroke survivors (Park et al., 2020); the non-robotic group improved more in movement smoothness and functional/impairment outcomes, whereas the robotic group improved more in movement speed. Physical robotic hand assistance showed a non-significant trend toward greater impairment recovery than virtual assistance, while virtual assistance significantly improved functional recovery (Farrens et al., 2025a).

High- and low-assistance robotic hand training improved impairment and function without significant between-group differences, although high assistance showed trends toward greater impairment recovery, pinch strength, and motivation, particularly in more severely impaired participants (Rowe et al., 2017). Assist-as-needed training produced significantly greater impairment and functional gains than constant guidance during shoulder-elbow rehabilitation (Cho and Song, 2019), whereas assist-as-needed and fixed subject-triggered control showed no significant difference in chronic incomplete spinal cord injury (Frullo et al., 2017).

Deficit versus null force field training also showed no significant difference (Wright et al., 2018), while velocity-dependent negative-viscosity training improved elbow movement performance (Huang and Patton, 2013). Progressive arm weight support, even without active robotic assistance, produced greater improvements in movement velocity, strength, and work area than full arm weight support (Ellis et al., 2009).

Movement-intention-contingent robotic assistance produced superior arm impairment recovery than non-contingent assistance in severely affected chronic stroke survivors (Ramos-Murguialday et al., 2013). However, a similar motor-imagery-based hand-training protocol improved both groups without significant between-group differences (Frolov et al., 2017); only the experimental group remained significant when chronic and subacute data were analyzed separately.

Overall, this qualitative summary highlights the contrasting findings across studies in which only a few studies have shown a positive effect of robotic assistance, and the rest are equivocal.

#### Risk of bias (ROB) analysis

The ROB analysis (Figure 2) revealed that 31% (4 of 13 studies) of trials have low ROB and 69% (9 out of 13 studies) of trials have some concern, mostly due to lack of double-blinding, potentially compromising the validity of the study-specific results.

**Figure 2:**
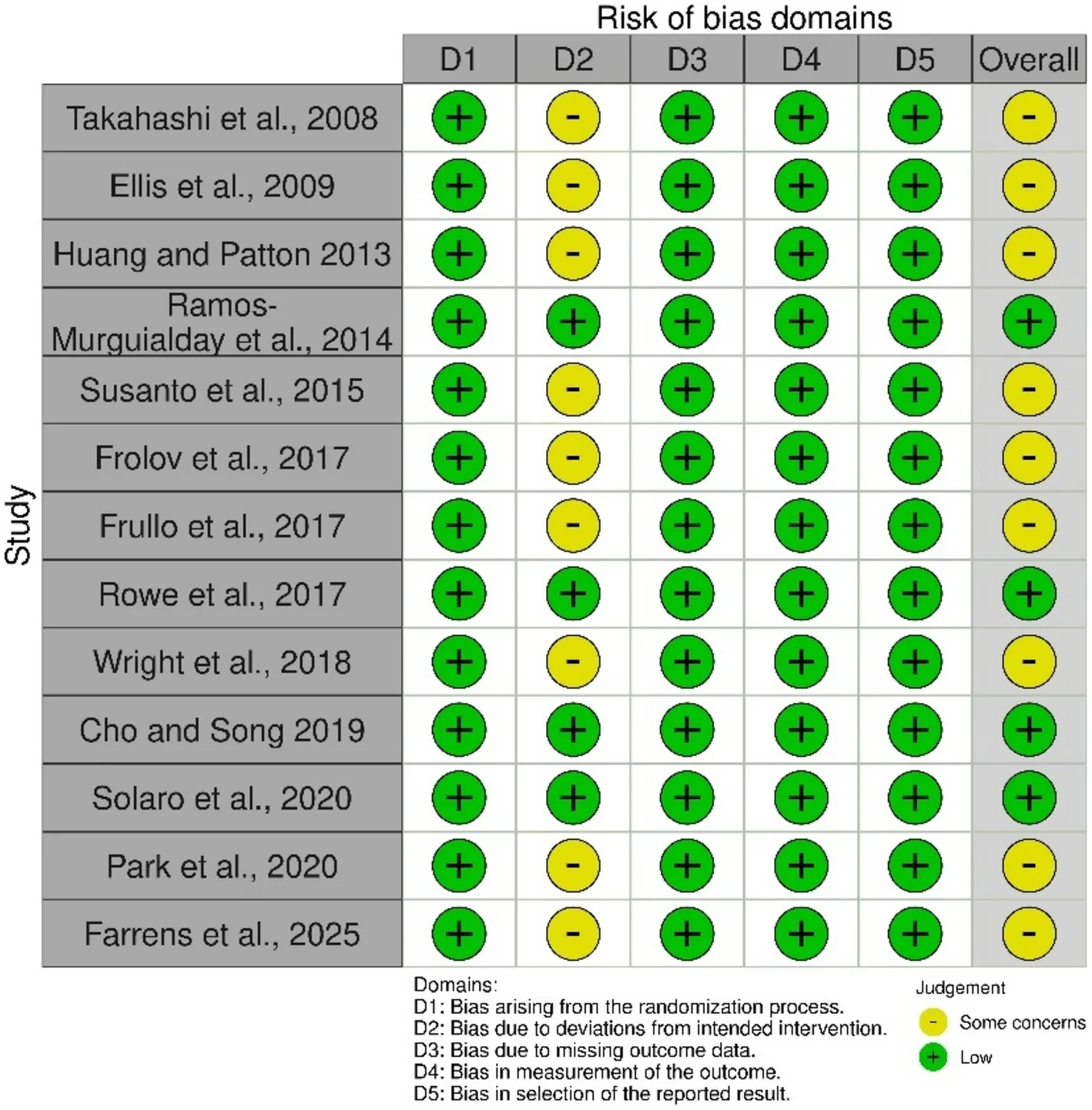
Risk of Bias analysis report of 13 included studies.

### Quantitative synthesis

The main analysis was performed on the pre-to-post intervention FMA scores pooled from nine controlled trials. The main analysis involved data from all nine trials, and subgroup analyses involved data from three to eight trials based on the specific aim of each analysis.

#### Main meta-analysis

The consolidated outcomes of the main and subgroup meta-analyses, summarized using both fixed-effect and random-effect models, demonstrated effect sizes within the range of 0.201 to 0.53, indicating small to moderate effects of intervention. The corresponding heterogeneity analyses revealed 20.57 to 62.62% between-study variability ( $I^{2}$ ), with the majority ≥ 45%, suggesting that the results should be interpreted with caution. Potential factors contributing to this substantial heterogeneity include inter-study differences in sample sizes, sample demographics, disease severity levels, robotic devices, movements/tasks trained, methodology, type of robotic assistance, and presence/absence of additional conventional therapy (these details can be found in Table 2 and Section V of the Supplementary material). Furthermore, as previously noted, the lack of double-blinding poses a significant risk of bias.

A significant fixed-effect summary (0.371 [0.116, 0.626]; *p* = 0.004) and non-significant random-effect summary (0.425 [−0.007, 0.858]; *p* = 0.053) were found for FMA score from the main meta-analysis involving 9 trials and 272 participants along with significant heterogeneity (62.62%, *p* = 0.006). The pooled estimate showed a small positive average effect favoring the experimental robot-assisted condition regardless of the stage of the disease, mode of the assistance, level of the assistance, and the targeted limb segment (Figure 3A; Table 4, row 2). However, the random-effects confidence interval included little or no effect, indicating considerable uncertainty regarding the magnitude and clinical relevance of this benefit. Individual study effects varied substantially, ranging from effects favoring the comparator to large effects favoring the experimental condition; therefore, the pooled effect was not driven uniformly across the evidence base and should be interpreted cautiously.

**Figure 3:**
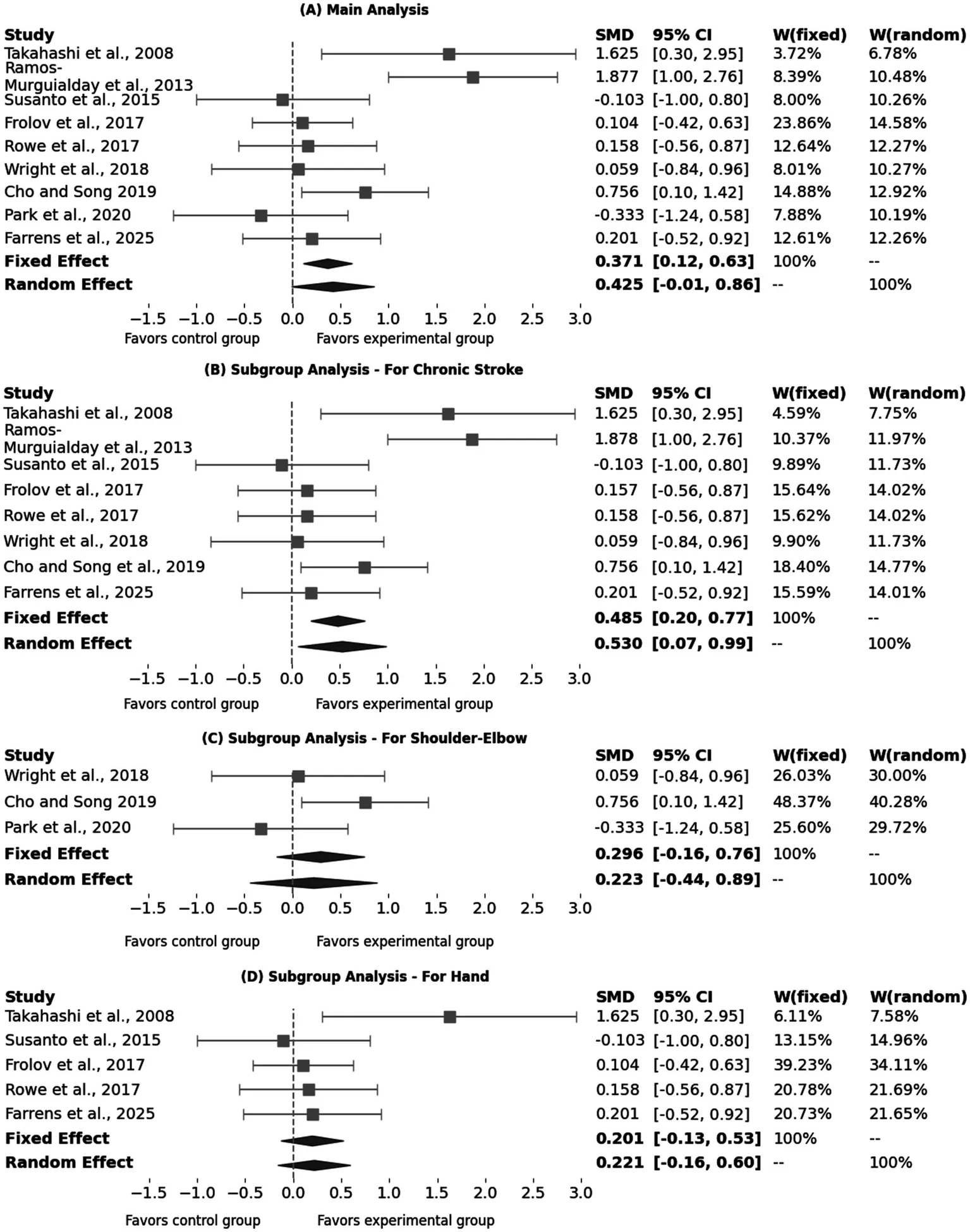
Forest plots representing multiple meta-analysis reports on FMA scores for **(A)** overall effect of robotic therapy, **(B)** chronic stroke, **(C)** therapy targeting shoulder-elbow, and **(D)** therapy targeting hand. For each study, the standardized mean difference (SMD) of FMA score with its 95% confidence interval (CI; rounded off to 2nd decimal place) and weights (W) for fixed-effect and random-effect models are given.

**Table 4:** Meta-analyses reports.

Analysis	FE-SMD with 95% CI	*p*-value -FE	RE-SMD with 95% CI	*p*-value -RE	df	Q	$\tau^{2}$	$I^{2}$ (%)	*p*-value—Q
Main analysis	0.371 [0.116, 0.626]	0.004	0.425 [−0.007, 0.858]	0.053	8	21.40	0.263	62.62	0.006
Subgroup analyses									
For chronic stroke	0.485 [0.201, 0.768]	0.0008	0.53 [0.067, 0.992]	0.0247	7	17.82	0.263	60.72	0.013
For shoulder-elbow	0.296 [−0.164, 0.755]	0.206	0.223 [−0.44, 0.887]	0.509	2	3.97	0.171	49.60	0.137
For hand	0.201 [−0.126, 0.528]	0.227	0.221 [−0.158, 0.601]	0.253	4	5.036	0.039	20.57	0.283

FE, fixed effect; RE, random effect; SMD, standardized mean difference; CI, confidence interval; df, degrees of freedom (i.e., total number of study −1).

Visual inspection of the main forest plot (Figure 3A) indicates that heterogeneity in the main analysis was driven disproportionately by two studies (Ramos-Murguialday et al., 2013; Takahashi et al., 2008), which reported markedly larger effect sizes (SMD = 1.877 and 1.625, respectively) than the remaining seven studies (SMD range: −0.333 to 0.756). Both outlying studies involved severely affected chronic stroke survivors receiving intensive, contingent forms of assistance (active hand assistance and BCI-contingent assistance, respectively) delivered with close supervision, which may plausibly explain their larger effects relative to the rest of the pool. Beyond these two studies, the remaining between-study variability likely reflects differences in robotic devices, rehabilitation protocols, treatment intensity, participant characteristics, and post-stroke stage, consistent with the qualitative heterogeneity in study design summarized in Table 2. Therefore, the overall pooled estimate should not be interpreted as representing a uniform treatment effect across different patient populations or robot-assisted strategies.

#### Subgroup analysis

The subgroup analysis of studies involving chronic stroke survivors (8 trials, 219 participants) showed a significant summary effect (fixed-effect: 0.485 [0.201, 0.768]; *p* = 0.0008; random-effect: 0.53 [0.067, 0.992]; *p* = 0.0247), suggesting a potential moderate benefit of robotic assistance on upper-limb impairment recovery in chronic stroke (Figure 3B; Table 4, row 4). However, the wide confidence interval and substantial heterogeneity (60.72%; *p* = 0.013) indicate considerable uncertainty regarding the magnitude and generalizability of this effect. Therefore, this subgroup finding should be regarded as suggestive rather than definitive.

Furthermore, the subgroup analyses for shoulder-elbow training (3 trials, 76 participants) and hand training (5 trials, 166 participants) showed non-significant pooled effects (Figure 3C; Table 4 row 5 and Figure 3D; Table 4 row 6 respectively). The random-effects point estimates were similar (0.223 and 0.221 for shoulder-elbow and hand, respectively); however, the corresponding confidence intervals were wide (shoulder-elbow [−0.44, 0.887]; hand [−0.158, 0.601]). Therefore, although the point estimates were numerically similar, the available evidence is insufficient to conclude that robotic assistance has equivalent effects for proximal and distal upper-limb training.

#### Publication bias

The assessment of publication bias revealed a mild asymmetry (visually) in the funnel plot, with some tendency for smaller, less precise studies to report larger positive effect sizes; however, eight of nine studies remained within the pseudo-95% confidence limits, and Egger’s regression test yielded an intercept (2.124) with *p* = 0.389, indicating no statistically significant evidence of funnel plot asymmetry or small-study effects. Nevertheless, given the limited number of studies included in the analysis, the possibility of publication bias cannot be excluded, and these findings should be interpreted cautiously. The funnel plot and additional information about this result are provided in Section IV (A) of the Supplementary material.

#### Sensitivity analysis

The sensitivity analysis showed that incorporating data of an additional study or changing the assumed pre-to-post measurement correlation coefficient of the included studies did not alter the direction of the pooled-effect estimates, whereas the magnitude, precision, and heterogeneity of the effects varied. The corresponding forest plots and parameter table can be found in Section IV (B) of the Supplementary material.

#### Meta-regression analysis

Exploratory univariable random-effect meta-regression analysis did not identify clear study-level moderators of the intervention effect. Three of the four investigated moderators did not explain the variation in pooled effects. Disease stage showed only a small explanatory contribution (R^2^ = 7.3%, *p* = 0.239), while severity and dosage revealed no explanatory value (R^2^ = 0% with *p* = 0.727 and *p* = 0.949, respectively). There was also no indication of a linear dose–response effect. Limb segment as a moderator showed a potentially important effect (R^2^ = 95%, *p* = 0.041), with the highest SMD observed for whole-arm interventions. However, because the analysis contains only a small number of studies and only one study for the whole-arm category, this result should be considered an exploratory moderator effect, rather than strong evidence of effect modification. Associated figures and additional information about these results are provided in Section IV (C) of the Supplementary material.

Overall, the quantitative findings indicate a small-to-moderate tendency favoring robotic assistance, but the strength of this inference is limited by substantial between-study heterogeneity, wide confidence intervals (imprecision), small sample sizes, and variability in participant and intervention characteristics. Sensitivity analyses showed that the direction of the pooled estimate was generally stable across alternative analytical assumptions, while its magnitude and precision varied. No statistically significant evidence of publication bias was detected; however, the small number of included studies limits the reliability of this assessment. Thus, the results show a possible beneficial contribution of robotic assistance to motor impairment recovery, but the magnitude and generalizability of this effect remain uncertain.

### Certainty of evidence

Consistent with the uncertainty observed in the quantitative synthesis, the overall GRADE certainty of evidence for the primary outcome measure (i.e. FMA score) was judged to be low. The randomized evidence was downgraded by one level for risk of bias, as several contributing studies raised methodological concerns, particularly the lack of double-blinding and small sample sizes. A further downgrade was applied (a) for inconsistency, owing to substantial variation in the magnitude and direction of individual study effects, with $I^{2}$ exceeding 60%, and (b) for imprecision, as the pooled random-effects confidence interval was wide and encompassed both little or no effect and a potentially clinically significant benefit. No downgrade was applied for indirectness, as the study populations, intervention contrasts, and impairment outcomes broadly aligned with the review question.

## Discussion

The findings of this systematic review and meta-analysis highlight the potential beneficial effect of robotic assistance on upper-limb sensorimotor recovery, particularly after stroke. The qualitative synthesis of 13 RCTs and the quantitative analyses of 9 RCTs lend some support for the premise that physical assistance or guidance provided by the rehabilitation robots may contribute to recovery. However, no strong conclusions can be drawn from the available evidence because of the limited number of controlled trials, small sample sizes, substantial heterogeneity, and variability in intervention characteristics. These findings also highlight the complexity of determining the optimal level and mode of robotic assistance, given the variability in patient responses and intervention parameters.

### Main findings and their clinical relevance

The findings are broadly consistent with recent evidence indicating that robotic rehabilitation has heterogeneous rather than uniform effects. Boardsworth et al. found only a small average benefit of dose-matched robotic rehabilitation for upper-limb capacity while identifying several device and program characteristics—including the level of robotic assistance—as potential effect modifiers (Boardsworth et al., 2025). Their findings suggest that the question is no longer simply whether robotic rehabilitation works but which features of robotic rehabilitation contribute to its effectiveness. The present review addresses a complementary and more focused question by preferentially including trials in which experimental conditions differed principally in the presence, amount, or mode of robotic assistance. This provides a more direct assessment of assistance itself, although at the cost of a considerably smaller evidence base. Alvaro et al. further emphasized that robotic training modality may interact with the stage of post-stroke recovery (Alvaro et al., 2026). Their synthesis suggested that assistive strategies may have relevance in chronic stroke, while active-assistive strategies may be useful during the subacute phase. The positive point estimate observed in the chronic subgroup of the present review is compatible with this possibility but should not be regarded as confirmation of stage-specific efficacy because of the limited number of studies and small sample sizes.

Although the evidence remains preliminary, the findings have some potential clinical and practical implications. The similar pooled effect sizes observed for shoulder-elbow and hand-focused training do not provide strong evidence that one anatomical target should be prioritized over the other on the basis of expected benefit from robotic assistance alone. However, both estimates were imprecise, and equivalence between proximal and distal training strategies cannot be established from the present evidence. Device selection should therefore continue to be guided primarily by patient-specific impairment, rehabilitation goals, and available equipment.

Similarly, the larger pooled effect observed in studies involving chronic stroke suggests that robotic assistance may remain relevant beyond the early recovery period, but this observation should be interpreted cautiously because of substantial heterogeneity and the limited evidence base. The studies contributing some of the largest individual effects involved contingent or intention-linked assistance, such as BCI-triggered (Ramos-Murguialday et al., 2013) or effort-contingent (Takahashi et al., 2008) control. This observation is compatible with an assist-as-needed approach to future device design and clinical protocols; however, it should be regarded as hypothesis-generating rather than evidence of superiority of one assistance strategy. Overall, these potential implications should not be considered practice-changing given the low GRADE certainty of the available evidence.

### Source of heterogeneity

A major finding of the present review was the substantial between-study heterogeneity. Studies differed in participant severity, chronicity, robotic platform, targeted limb segment, assistance strategy, movement task, treatment intensity, feedback modality, and concomitant conventional therapy, all of which could plausibly influence the effect of robotic assistance. The detailed intervention review in Supplementary material (Section V) further showed that several potentially important protocol characteristics, including movement geometry, force levels, repetitions, progression of assistance, feedback schedules, and rest periods, were incompletely reported.

Visual inspection of the main forest plot suggested that heterogeneity was influenced disproportionately by Takahashi et al. (2008) and Ramos-Murguialday et al. (2013), which reported substantially larger effects than the remaining studies. Both involved severely affected individuals with chronic stroke who received intensive, contingent forms of assistance—active hand assistance and BCI-contingent assistance, respectively—which may have contributed to their larger effects. However, these characteristics cannot be considered established explanations for the observed effect sizes because multiple intervention and participant characteristics differed across studies.

Exploratory meta-regression analysis suggested that the targeted limb segment may partly explain the observed variability, whereas post-stroke stage, severity, and dosage did not appear to account for differences in effect size. However, given the small number of studies and sparse representation within some moderator categories, these findings should be considered exploratory. The persistence of unexplained heterogeneity for most examined moderators indicates that between-study variability is likely multifactorial and may reflect differences in robotic devices, rehabilitation protocols, assistance strategies, treatment intensity, participant characteristics, and post-stroke stage. Incomplete reporting of several intervention parameters further limits the ability to identify which specific protocol characteristics account for the remaining variability.

### Mechanistic rationale for robotic assistance

Robotic assistance enables high-repetition, high-dose training and may extend access to therapy (Rowe et al., 2017; Reinkensmeyer and Housman, 2007; Balasubramanian, 2026; Farrens et al., 2025b). It is integrated with technologies such as brain–computer interfaces (BCIs) to enhance engagement and sensorimotor feedback, particularly in individuals with severe impairment (Ramos-Murguialday et al., 2013; Alashram, 2025; Ren et al., 2024). These applications are relevant to the present review, which suggests that outcomes may depend not only on whether assistance is provided but also on how it is delivered, particularly whether it is contingent on the participant’s intention or effort.

A structural causal model of robot-assisted neurorehabilitation (Ellis et al., 2009) proposed two potential mechanisms: a challenge-motivation pathway and an enhanced sensory-feedback pathway. Robotic assistance can enable movements that may not be achievable voluntarily (De Iaco et al., 2024; Park et al., 2025; Boardsworth et al., 2025; Alvaro et al., 2026; Wang et al., 2025), thereby modulating task challenge and motivation (Guadagnoli and Lee, 2004). Farrens et al. (2025b) similarly reported greater intrinsic motivation with physical robotic assistance than with virtual or no assistance. Thus, an appropriate amount of robotic assistance that optimizes task challenge and motivation may improve sensorimotor outcomes by promoting more active participation in therapy. Robotic assistance may also enable larger and faster movements than voluntary movement alone (Hu et al., 2009; Murakami et al., 2023; Fasoli et al., 2003; Stein et al., 2004; Jeon et al., 2024; Liu et al., 2018; Balasubramanian and He, 2012; Zhang et al., 2022). Together with contact pressure and interaction forces at the human–machine interface, these movements may generate task-related sensory feedback. When temporally coupled to motor intent, such feedback may contribute to cortical changes through Hebbian-like learning (Ramos-Murguialday et al., 2013; Takahashi et al., 2008; Rowe et al., 2017; Banyai and Brișan, 2024; Huang and Krakauer, 2009). From this perspective, the therapeutic effect may depend on the coupling between robotic assistance, sensory feedback, voluntary effort, and motor intention.

Neuromuscular electrical stimulation (NMES) provides a related framework because it assists movement through peripheral neuromuscular activation (Ethier et al., 2015; Cordo et al., 2022; Carson and Buick, 2021; Popović et al., 2009) while generating substantial afferent feedback. Reviews (Eraifej et al., 2017; Howlett et al., 2015) report modest improvements in upper-limb impairment and activity when NMES is combined with voluntary effort, while another review (Bai et al., 2020) found greater improvement with BCI plus NMES than with BCI plus robotic assistance, supporting a tentative role for enhanced movement-related sensory feedback. Robotic assistance may also provide musculoskeletal benefits by enabling movement beyond the active range of motion and stretching antagonist muscles and periarticular structures (Ethier et al., 2015; Popović et al., 2009; Seeman et al., 2017). However, relatively short-term stretching protocols, including interventions of approximately three months, did not significantly improve joint range of motion (Salazar et al., 2019), suggesting that the contribution of musculoskeletal effects to functional recovery remains limited.

Overall, these mechanisms provide plausible pathways through which the timing, magnitude, and contingency of robotic assistance may influence sensorimotor recovery. However, they should be regarded as hypotheses requiring direct experimental testing rather than established explanations for the pooled effects observed in this meta-analysis. Future trials should systematically manipulate assistance level, effort contingency, sensory-feedback characteristics, and patient engagement while controlling other therapy components to determine which mechanisms contribute most strongly to recovery.

### Limitations and implications for future research

Several limitations should be considered when interpreting the findings. The present review focused specifically on RCTs in which groups differed in the presence, level, or mode of robotic assistance and was restricted to upper-limb rehabilitation. Consequently, the findings do not address other forms of human–robot physical interaction, including assistive versus resistive training, error-augmenting versus error-reduction training, and active versus passive training. Studies involving lower-limb or whole-body rehabilitation were also not considered. In addition, this review did not examine the broader effect of robotic assistance on functional recovery or activities of daily living.

The limited number of studies also constrained the exploration of between-study heterogeneity. Although meta-regression analysis was performed for selected moderators, the small number of studies and sparse representation within several categories limited statistical power, and the resulting associations should therefore be considered exploratory. In addition, heterogeneity related to device characteristics, assistance modalities, and other intervention-specific parameters could not be examined robustly because of substantial protocol variability and incomplete reporting across studies. Consequently, a considerable proportion of the observed heterogeneity remains unexplained.

Future studies should therefore use adequately powered, randomized designs that isolate the contribution of physical assistance while carefully matching other intervention components between the groups. More consistent reporting of assistance magnitude, control strategy, progression rules, movement geometry, treatment dose, feedback, repetitions, and rest periods would facilitate comparison across studies and enable more robust examination of potential effect modifiers. Studies should also examine whether the response to robotic assistance varies systematically with participant characteristics, stage of recovery, impairment severity, anatomical target, and mode of assistance. Such work is necessary to determine not only whether robotic assistance contributes independently to recovery but also which assistance strategies are most appropriate for particular patient populations.

## Conclusion

This study investigated the role of robotic assistance in sensorimotor recovery through a systematic review and meta-analysis of RCTs on upper-limb robotic rehabilitation. The findings are best characterized as preliminary and fragmented rather than conclusive: They provide encouraging, hypothesis-generating evidence that the presence of robotic assistance may improve upper-limb impairment outcomes after neurological disorders, particularly stroke, relative to unassisted training—under otherwise matched conditions, but do not yet constitute definitive evidence of an independent or mechanistic benefit.

The limited number of appropriately designed studies, risk of bias concerns, small sample sizes, substantial heterogeneity, wide confidence intervals around the pooled effects, and methodological variability across studies limit the stability and generalizability of the current estimates. Differences in intervention protocols, assistance strategies, participant characteristics, and outcome measures may also have contributed to the observed variability. Therefore, the available evidence does not permit firm conclusions regarding the independent contribution of robotic assistance to sensorimotor recovery.

This study nevertheless highlights the dearth of rigorous, sufficiently powered studies evaluating the clinical value of the physical assistance delivered by a robot during therapy. Despite significant investment in the development of diverse robotic systems (over 38 types) (De Iaco et al., 2024) for upper-limb neurorehabilitation, the independent contribution of robotic assistance to recovery remains poorly understood. A recent systematic review and meta-analysis by De Iaco et al. also emphasized the need to focus on the mechanistic basis of robot-assisted therapy (De Iaco et al., 2024). Fundamentally, without physical assistance, a robot functions primarily as a sensing and feedback tool. Therefore, isolating and quantifying the role of robotic assistance is essential. Overall, there is an urgent need for larger, adequately powered, high-quality RCTs with standardized CONSORT-compliant protocol (Hart et al., 2019; Schulz et al., 2010) to determine whether the encouraging effects observed here reflect a true independent contribution of robotic assistance to recovery.

## Data Availability

The original contributions presented in the study are included in the article/Supplementary material, further inquiries can be directed to the corresponding author.

## References

[ref1] Alashram A. R. (2025). Combined robot-assisted therapy and neuromuscular electrical stimulation in upper limb rehabilitation in patients with stroke: a systematic review of randomized controlled trials. J. Hand Ther. 38, 347–359. doi: 10.1016/j.jht.2025.04.002, 40615305

[ref2] Alvaro F. Perrelli M. Adduci R. Tedesco F. Mundo D. (2026). On the correlation between training modalities and recovery stages in poststroke robotic rehabilitation of the upper limb: a systematic review. J. Neuroeng. Rehabil. 23:197. doi: 10.1186/s12984-026-02005-7, 42063147 PMC13285516

[ref3] Bai Z. Fong K. N. K. Zhang J. J. Chan J. Ting K. H. (2020). Immediate and long-term effects of BCI-based rehabilitation of the upper extremity after stroke: a systematic review and meta-analysis. J. Neuroeng. Rehabil. 17:57. doi: 10.1186/s12984-020-00686-2, 32334608 PMC7183617

[ref4] Balasubramanian S. (2026). A structural causal model for robot-assisted upper-limb neurorehabilitation. Front Rehabil Sci. 7:1819379. doi: 10.3389/fresc.2026.1819379, 42523754 PMC13407635

[ref5] Balasubramanian S. He J. (2012). Adaptive control of a wearable exoskeleton for upper-extremity neurorehabilitation. Appl. Bionics Biomech. doi: 10.3233/ABB-2011-0041

[ref6] Banyai A. D. Brișan C. (2024). Robotics in physical rehabilitation: systematic review. Healthcare. 12:1720. doi: 10.3390/healthcare12171720, 39273744 PMC11395122

[ref7] Bertani R. Melegari C. De Cola M. C. Bramanti A. Bramanti P. Calabrò R. S. (2017). Effects of robot-assisted upper limb rehabilitation in stroke patients: a systematic review with meta-analysis. Neurol. Sci. 38, 1561–1569. doi: 10.1007/s10072-017-2995-5, 28540536

[ref8] Boardsworth K. Rashid U. Olsen S. Rodriguez-Ramirez E. Browne W. Alder G. . (2025). Upper limb robotic rehabilitation following stroke: a systematic review and meta-analysis investigating efficacy and the influence of device features and program parameters. J. Neuroeng. Rehabil. 22:164. doi: 10.1186/s12984-025-01662-4, 40671078 PMC12265130

[ref9] Bouchard A. E. Corriveau H. Milot M. H. (2017). A single robotic session that guides or increases movement error in survivors’ post-chronic stroke: which intervention is best to boost the learning of a timing task? Disabil. Rehabil. 39, 1607–1614. doi: 10.1080/09638288.2016.1205151 27415452

[ref10] Carson R. G. Buick A. R. (2021). Neuromuscular electrical stimulation-promoted plasticity of the human brain. J. Physiol. 599, 2375–2399. doi: 10.1113/JP278298, 31495924

[ref11] Cho K. H. Song W. K. (2019). Robot-assisted reach training with an active assistant protocol for long-term upper extremity impairment poststroke: a randomized controlled trial. Arch. Phys. Med. Rehabil. 100, 213–219. doi: 10.1016/j.apmr.2018.10.002 30686326

[ref12] Cohen J. (1988). Statistical Power Analysis for the Behavioral Sciences. 2nd Edn Hillsdale, NJ: Lawrence Erlbaum Associates.

[ref13] Cordo P. Wolf S. Rymer W. Z. Byl N. Stanek K. Hayes J. R. (2022). Assisted movement with proprioceptive stimulation augments recovery from moderate-to-severe upper limb impairment during subacute stroke period: a randomized clinical trial. Neurorehabil. Neural Repair 36, 239–250. doi: 10.1177/15459683211063159, 35067125

[ref14] Covidence. (2014). Systematic Review Software, Veritas Health Innovation, Melbourne, Australia. Available online at: www.covidence.org

[ref15] De Iaco L. Veerbeek J. M. Ket J. C. F. Kwakkel G. (2024). Upper limb robots for recovery of motor arm function in patients with stroke: a systematic review and Meta-analysis. Neurology 103:e209495. doi: 10.1212/WNL.0000000000209495, 38870442

[ref16] Deeks J. J. Higgins J. P. T. Altman D. G. McKenzie J. E. Veroniki A. A. (2024). “Chapter 10: Analysing data and undertaking meta-analyses,” in Cochrane Handbook for Systematic Reviews of Interventions, eds. Higgins J. P. T. Thomas J. Chandler J. (Chichester (UK): John Wiley & Sons).

[ref17] Ellis M. D. Sukal-Moulton T. Dewald J. P. A. (2009). Progressive shoulder abduction loading is a crucial element of arm rehabilitation in chronic stroke. Neurorehabil. Neural Repair 23, 862–869. doi: 10.1177/1545968309332927, 19454622 PMC2833097

[ref18] Eraifej J. Clark W. France B. Desando S. Moore D. (2017). Effectiveness of upper limb functional electrical stimulation after stroke for the improvement of activities of daily living and motor function: a systematic review and meta-analysis. Syst. Rev. 6:40. doi: 10.1186/s13643-017-0435-5, 28245858 PMC5331643

[ref19] Ethier C. Gallego J. A. Miller L. E. (2015). Brain-controlled neuromuscular stimulation to drive neural plasticity and functional recovery. Curr. Opin. Neurobiol. 33, 95–102. doi: 10.1016/j.conb.2015.03.007, 25827275 PMC4523462

[ref20] Farrens A. J. Garcia-Fernandez L. Roja D. R. Estrada J. O. Reinsdorf D. Chan V. . (2025a). Tailored robotic training improves hand function and proprioceptive processing in stroke survivors with proprioceptive deficits: a randomized controlled trial. arXiv. doi: 10.48550/arXiv.2511.00259

[ref21] Farrens A. J. Garcia-Fernandez L. Rojas R. D. Estrada J. O. Reinsdorf D. Chan V. . (2025b). Variants of active assist robotic therapy: feasibility of virtual assistance and proprioceptive training as gauged by their effects on success and motivation during finger movement training after stroke. J. Neuroeng. Rehabil. 22:167. doi: 10.1186/s12984-025-01667-z, 40682190 PMC12273468

[ref22] Fasoli S. E. Krebs H. I. Stein J. Frontera W. R. Hogan N. (2003). Effects of robotic therapy on motor impairment and recovery in chronic stroke. Arch. Phys. Med. Rehabil. 84, 477–482. doi: 10.1053/apmr.2003.50110, 12690583

[ref23] Frolov A. A. Mokienko O. Lyukmanov R. Biryukova E. Kotov S. Turbina L. . (2017). Post-stroke rehabilitation training with a motor-imagery-based brain-computer interface (BCI)-controlled hand exoskeleton: a randomized controlled multicentre trial. Front. Neurosci. 11:400. doi: 10.3389/fnins.2017.00400 PMC5517482 28775677

[ref24] Frullo J. M. Elinger J. Pehlivan A. U. Fitle K. Nedley K. Francisco G. E. . (2017). Effects of assist-as-needed upper extremity robotic therapy after incomplete spinal cord injury: a parallel-group controlled trial. Front. Neurorobot. 11:26. doi: 10.3389/fnbot.2017.00026, 28659784 PMC5469353

[ref25] Givon-Mayo R. Simons E. Ohry A. Karpin H. Israely S. Carmeli E. (2014). A preliminary investigation of error enhancement of the velocity component in stroke patients' reaching movements. Int. J. Ther. Rehabil. 21, 160–168. doi: 10.12968/ijtr.2014.21.4.160

[ref26] Guadagnoli M. A. Lee T. D. (2004). Challenge point: a framework for conceptualizing the effects of various practice conditions in motor learning. J. Mot. Behav. 36, 212–224. doi: 10.3200/JMBR.36.2.212-224, 15130871

[ref27] Hart T. Dijkers M. P. Whyte J. Turkstra L. S. Zanca J. M. Packel A. . (2019). A theory-driven system for the specification of rehabilitation treatments. Arch. Phys. Med. Rehabil. 100, 172–180. doi: 10.1016/j.apmr.2018.09.109, 30267669

[ref28] Higgins J. P. T. Savović J. Page M. J. Elbers R. G. Sterne J. A. C.. Chapter 8: assessing risk of bias in a randomized trial. In: Higgins J. P. T. Thomas J. Chandler J. Cumpston M. Li T. Page M. J. Cochrane Handbook for Systematic Reviews of Interventions. Version 6.5. Cochrane; (2024). Chichester (UK): John Wiley & Sons, (Accessed April 11, 2025). Available online at: https://www.training.cochrane.org/handbook.

[ref29] Hogan N. Krebs H. I. Rohrer B. Palazzolo J. J. Dipietro L. Fasoli S. E. . (2006). Motions or muscles? Some behavioral factors underlying robotic assistance of motor recovery. J. Rehabil. Res. Dev. 43:605. doi: 10.1682/JRRD.2005.06.0103, 17123202

[ref30] Howlett O. A. Lannin N. A. Ada L. Mckinstry C. (2015). Functional electrical stimulation improves activity after stroke: a systematic review with meta-analysis. Arch. Phys. Med. Rehabil. 96, 934–943. doi: 10.1016/j.apmr.2015.01.013, 25634620

[ref31] Hu X. L. Tong K. Y. Song R. Zheng X. J. Leung W. W. F. (2009). A comparison between electromyography-driven robot and passive motion device on wrist rehabilitation for chronic stroke. Neurorehabil. Neural Repair 23, 837–846. doi: 10.1177/1545968309338191, 19531605

[ref32] Huang V. S. Krakauer J. W. (2009). Robotic neurorehabilitation: a computational motor learning perspective. J. Neuroeng. Rehabil. 6:5. doi: 10.1186/1743-0003-6-5, 19243614 PMC2653497

[ref33] Huang F. C. Patton J. L. (2013). Augmented dynamics and motor exploration as training for stroke. I.E.E.E. Trans. Biomed. Eng. 60, 838–844. doi: 10.1109/TBME.2012.2192116, 22481803 PMC4914037

[ref34] Jeon S. Y. Ki M. Shin J. H. (2024). Resistive versus active assisted robotic training for the upper limb after a stroke: a randomized controlled study. Ann. Phys. Rehabil. Med. 67:101789. doi: 10.1016/j.rehab.2023.101789, 38118340

[ref35] Krebs H. I. Palazzolo J. J. Dipietro L. Ferraro M. Krol J. Rannekleiv K. . (2003). Rehabilitation robotics: performance-based progressive robot-assisted therapy. Auton. Robot. 15, 7–20. doi: 10.1023/A:1024494031121

[ref36] Lee B. O. Saragih I. D. Batubara S. O. (2023). Robotic arm use for upper limb rehabilitation after stroke: a systematic review and meta-analysis. Kaohsiung J. Med. Sci. 39, 435–445. doi: 10.1002/kjm2.12679, 36999894 PMC11895899

[ref37] Lin L. Aloe A. M. (2021). Evaluation of various estimators for standardized mean difference in meta-analysis. Stat. Med. 40, 403–426. doi: 10.1002/sim.8781, 33180373 PMC7770064

[ref38] Liu L. Y. Li Y. L. Lamontagne A. (2018). The effects of error-augmentation versus error-reduction paradigms in robotic therapy to enhance upper extremity performance and recovery post-stroke: a systematic review. J. Neuroeng. Rehabil. 15:65. doi: 10.1186/s12984-018-0408-5, 29973250 PMC6033222

[ref39] Lo A. C. Guarino P. D. Richards L. G. Haselkorn J. K. Wittenberg G. F. Federman D. G. . (2010). Robot-assisted therapy for long-term upper-limb impairment after stroke. N. Engl. J. Med. 362, 1772–1783. doi: 10.1056/nejmoa0911341, 20400552 PMC5592692

[ref40] Maciejasz P. Eschweiler J. Gerlach-Hahn K. Jansen-Troy A. Leonhardt S. (2014). A survey on robotic devices for upper limb rehabilitation. J. Neuroeng. Rehabil. Rev. 11:3. doi: 10.1186/1743-0003-11-3 PMC4029785 24401110

[ref41] Marchal-Crespo L. Reinkensmeyer D. J. (2009). Review of control strategies for robotic movement training after neurologic injury. J. Neuroeng. Rehabil. 6. doi: 10.1186/1743-0003-6-20, 19531254 PMC2710333

[ref42] McGuinness L. A. Higgins J. P. T. (2021). Risk-of-Bias VISualization (robvis): an R package and shiny web app for visualizing risk-of-bias assessments. Res. Synth. Methods 12, 55–61. doi: 10.1002/jrsm.1411, 32336025

[ref43] Mehrholz J. Pohl M. Platz T. Kugler J. Elsner B. (2018). Electromechanical and robot-assisted arm training for improving activities of daily living, arm function, and arm muscle strength after stroke. Cochrane Database Syst. Rev. 9:CD006876. doi: 10.1002/14651858.CD006876.pub5, 30175845 PMC6513114

[ref44] Murakami Y. Honaga K. Kono H. Haruyama K. Yamaguchi T. Tani M. . (2023). New artificial intelligence-integrated electromyography-driven robot hand for upper extremity rehabilitation of patients with stroke: a randomized, controlled trial. Neurorehabil. Neural Repair 37, 298–306. doi: 10.1177/15459683231166939 37039319

[ref45] Page M. J. McKenzie J. E. Bossuyt P. M. Boutron I. Hoffmann T. C. Mulrow C. D. . (2020). The PRISMA 2020 statement: an updated guideline for reporting systematic reviews. BMJ 372:n71. doi: 10.1136/bmj.n71, 33782057 PMC8005924

[ref46] Park J. H. Park G. Kim H. Y. Lee J. Y. Ham Y. Hwang D. . (2020). A comparison of the effects and usability of two exoskeletal robots with and without robotic actuation for upper extremity rehabilitation among patients with stroke: a single-blinded randomised controlled pilot study. J. Neuroeng. Rehabil. 17:137. doi: 10.1186/s12984-020-00763-6, 33076952 PMC7574181

[ref47] Park J. M. Park H. J. Yoon S. Y. Kim Y. W. Shin J. I. Lee S. C. (2025). Effects of robot-assisted therapy for upper limb rehabilitation after stroke: an umbrella review of systematic reviews. Stroke 56, 1243–1252. doi: 10.1161/STROKEAHA.124.048183, 40115991

[ref48] Pila O. Koeppel T. Duret C. Jamin P. (2021). Comparison of active-assisted and active-unassisted robot-mediated upper limb therapy in subacute stroke. Restor. Neurol. Neurosci. 39, 1–7. doi: 10.3233/RNN-201010, 33285649

[ref49] Popović D. B. Sinkjær T. Popović M. B. (2009). Electrical stimulation as a means for achieving recovery of function in stroke patients. NeuroRehabilitation 25, 45–58. doi: 10.3233/NRE-2009-0498, 19713618

[ref50] Ramos-Murguialday A. Broetz D. Rea M. Läer L. Yilmaz Ö. Brasil F. L. . (2013). Brain-machine interface in chronic stroke rehabilitation: a controlled study. Ann. Neurol. 74, 100–108. doi: 10.1002/ana.23879, 23494615 PMC3700597

[ref51] Reinkensmeyer D. J. Housman S. J. (2007). “If I can't do it once, why do it a hundred times?”: connecting volition to movement success in a virtual environment motivates people to exercise the arm after stroke,” in 2007 Virtual Rehabilitation, (Venice, Italy: IEEE), 44–48.

[ref52] Reinkensmeyer D. J. Takahashi C. D. Timoszyk W. K. Reinkensmeyer A. N. Kahn L. E. (2001). Design of robot assistance for arm movement therapy following stroke. Adv. Robot. 14, 625–637. doi: 10.1163/156855301742058

[ref53] Ren C. Li X. Gao Q. Pan M. Wang J. Yang F. . (2024). The effect of brain-computer interface controlled functional electrical stimulation training on rehabilitation of upper limb after stroke: a systematic review and meta-analysis. Front. Hum. Neurosci. 18:1438095. doi: 10.3389/fnhum.2024.1438095, 39391265 PMC11464471

[ref54] Rohatgi A. (2011). WebPlotDigitizer. Available online at: https://automeris.io (Accessed August, 2024).

[ref55] Rowe J. B. Chan V. Ingemanson M. L. Cramer S. C. Wolbrecht E. T. Reinkensmeyer D. J. (2017). Robotic assistance for training finger movement using a Hebbian model: a randomized controlled trial. Neurorehabil. Neural Repair 31, 769–780. doi: 10.1177/1545968317721975, 28803535 PMC5894506

[ref56] Salazar A. P. Pinto C. Ruschel Mossi J. V. Figueiro B. Lukrafka J. L. Pagnussat A. S. (2019). Effectiveness of static stretching positioning on post-stroke upper-limb spasticity and mobility: systematic review with meta-analysis. Ann. Phys. Rehabil. Med. 62, 274–282. doi: 10.1016/j.rehab.2018.11.004, 30582986

[ref57] Schulz K. F. Altman D. G. Moher D. (2010). CONSORT 2010 statement: updated guidelines for reporting parallel group randomised trials. BMC Med. 8:18. doi: 10.1186/1741-7015-8-18, 20334633 PMC2860339

[ref58] Schünemann H. J. Higgins J. P. T. Vist G. E. Glasziou P. Akl E. A. Skoetz N. . (2019). “Chapter 14: completing “summary of findings” tables and grading the certainty of the evidence,” in Cochrane Handbook for Systematic Reviews of Interventions. Chichester (UK): John Wiley & Sons.

[ref59] Seeman S. C. Mogen B. J. Fetz E. E. Perlmutter S. I. (2017). Paired stimulation for spike-timing-dependent plasticity in primate sensorimotor cortex. J. Neurosci. 37, 1935–1949. doi: 10.1523/JNEUROSCI.2046-16.2017, 28093479 PMC5320619

[ref60] Solaro C. Cattaneo D. Basteris A. Carpinella I. de Luca A. Mueller M. . (2020). Haptic vs sensorimotor training in the treatment of upper limb dysfunction in multiple sclerosis: a multi-center, randomised controlled trial. J. Neurol. Sci. 412:116743. doi: 10.1016/j.jns.2020.116743, 32145522

[ref61] Stein J. Krebs H. I. Frontera W. R. Fasoli S. E. Hughes R. Hogan N. (2004). Comparison of two techniques of robot-aided upper limb exercise training after stroke. Am. J. Phys. Med. Rehabil. 83, 720–728. doi: 10.1097/01.PHM.0000137313.14480.CE, 15314537

[ref62] Susanto E. A. Tong R. K. Y. Ockenfeld C. Ho N. S. K. (2015). Efficacy of robot-assisted fingers training in chronic stroke survivors: a pilot randomized-controlled trial. J. Neuroeng. Rehabil. 12:42. doi: 10.1186/s12984-015-0033-5, 25906983 PMC4422529

[ref63] Takahashi C. D. Der-Yeghiaian L. Le V. Motiwala R. R. Cramer S. C. (2008). Robot-based hand motor therapy after stroke. Brain 131, 425–437. doi: 10.1093/brain/awm311, 18156154

[ref64] Takahashi K. Domen K. Sakamoto T. Toshima M. Otaka Y. Seto M. . (2016). Efficacy of upper extremity robotic therapy in subacute Poststroke hemiplegia: an exploratory randomized trial. Stroke 47, 1385–1388. doi: 10.1161/STROKEAHA.115.012520, 27006452

[ref65] Takebayashi T. Takahashi K. Okita Y. Kubo H. Hachisuka K. Domen K. (2022). Impact of the robotic-assistance level on upper extremity function in stroke patients receiving adjunct robotic rehabilitation: sub-analysis of a randomized clinical trial. J. Neuroeng. Rehabil. 19:25. doi: 10.1186/s12984-022-00986-9, 35216603 PMC8881821

[ref66] Volpe B. Krebs H. Hogan N. Edelstein L. Diels C. Aisen M. (2000). A novel approach to stroke rehabilitation robot-aided sensorimotor stimulation. Neurology 54, 1938–1944. doi: 10.1212/WNL.54.10.1938, 10822433

[ref67] Wang L. Li X. Liu Y. Zhang E. Li C. (2025). Effects of robotic-assisted upper extremity therapy for stroke patients in different recovery phases: a systematic review and meta-analysis. Front. Neurol. 16:1640522. doi: 10.3389/fneur.2025.1640522, 41602970 PMC12832252

[ref68] Warraich Z. Kleim J. A. (2010). Neural plasticity: the biological substrate for neurorehabilitation. PM and R. 2, S208–S219. doi: 10.1016/j.pmrj.2010.10.016, 21172683

[ref69] Wright Z. A. Lazzaro E. Patton J. L. Huang F. C. (2018). Robot training with vector fields based on stroke survivors’ individual movement statistics. IEEE Trans. Neural Syst. Rehabil. Eng. 26, 307–323. doi: 10.1109/TNSRE.2017.2763458 PMC5901661 29035220

[ref70] Wright Z. A. Patton J. L. Huang F. C. Lazzaro E. (2015). “Evaluation of force field training customized according to individual movement deficit patterns,” in Presented at: 2015 IEEE International Conference on Rehabilitation Robotics (ICORR). 193–198.

[ref71] Yue Z. Zhang X. Wang J. (2017). Hand rehabilitation robotics on Poststroke motor recovery. Behav. Neurol. 2017, 1–20. doi: 10.1155/2017/3908135, 29230081 PMC5688261

[ref72] Zhang L. Guo S. Xi F. (2022). Performance-based assistance control for robot-mediated upper-limbs rehabilitation. Mechatronics. 89:102919. doi: 10.1016/j.mechatronics.2022.102919

