# Supplementary material for: Effect of robotic assistance on upper-limb sensorimotor recovery: a systematic review and meta-analysis
Source: Front Neurorobot. 2026 Oct 20;20:1860365. doi: 10.3389/fnbot.2026.1860365 (PMC13727536; doi:10.3389/fnbot.2026.1860365)
Supplement: Supplementary file 1 [file Data_Sheet_1.pdf]

Supplementary Material

Effect of robotic assistance on upper-limb sensorimotor recovery: a systematic review and meta-analysis

Ritwika Choudhuri[1,2], John M Solomon[3], Aravind Nehrujee[4,5], S. Sujatha[2] and Sivakumar Balasubramanian[1,6]

[1]Department of Bioengineering, Christian Medical College Vellore, Bagayam, Vellore, India
[2]Department of Mechanical Engineering, Indian Institute of Technology Madras, Chennai, India
[3]Department of Physiotherapy, Manipal College of Health Professions, Manipal Academy of Higher Education, Manipal, India
[4]Shirley Ryan AbilityLabs, Chicago, Illinois, USA
[5]Department of Physical Medicine and Rehabilitation, Northwestern University, Chicago, Illinois, USA
[6]School of Health & Rehabilitation Sciences, The University of Queensland, Brisbane, Australia
Corresponding author: Prof. Sivakumar Balasubramanian
Address: Department of Bioengineering, Christian Medical College Vellore, Bagayam, Vellore- 632002, Tamil Nadu, India. Email: siva82kb@cmcvellore.ac.in; Telephone: +91 416 2285098

Section-I:

Following search strategies were used to find relevant papers in electronic databases-

a) For advance search in PubMed-

1. ((Robot) OR (Robot Assisted) OR (Robotic Device) OR (End-Effector) OR (Exoskeleton) OR (Orthoses)) AND ((Active Assist) OR (Active Resist) OR (High Assistance) OR (Low Assistance) OR (Active Assistance) OR (Passive Assistance) OR (Unassisted) OR (Not Assisted)) AND ((Rehabilitation) OR (Neurorehabilitation) OR (Training) OR (Therapy) OR (Recovery)) AND ((Upper Limb) OR (Upper Extremity) OR (Hand) OR (Arm) OR (Shoulder-Elbow) OR (Wrist) OR (Finger)) AND (Control Group) AND (Patients) NOT (Review) NOT (Follow-up) NOT (Animal) NOT (Hip) NOT (Lower Limb) NOT (Leg) NOT (Knee) NOT (Foot) NOT (Gait) NOT (Surgery) NOT (Laparoscopy) NOT (Cancer)

2. ((Upper Limb) OR (Upper Extremity) OR (Hand) OR (Arm) OR (Shoulder-Elbow) OR (Wrist) OR (Finger)) AND ((Rehabilitation) OR (Training) OR (Therapy) OR (Recovery)) AND ((Robot) OR (Robotic Device) OR (Robot-assisted) OR (Robotic Assistance) OR (Robotic Training) OR (Robot-mediated) OR (Robot-based) OR (Robotic Rehabilitation) OR (End-effector) OR (Exoskeleton) OR (Orthoses)) AND ((Active Assisted) OR (Resistive) OR (High Assistance) OR

(Low Assistance) OR (Active) OR (Passive)) AND (Control Group) NOT ((Review) OR (Animal) OR (Hip) OR (Lower Limb) OR (Leg) OR (Knee) OR (Foot) OR (Gait) OR (Walking) OR (Surgery) OR (Laparoscopy) OR (Cancer))

3. ((Robot-assisted) OR (Robotic Assistance) OR (Robotic Training) OR (Robot-mediated) OR (Robot-based) OR (Robotic Rehabilitation) OR (End-effector) OR (Exoskeleton) OR (Orthoses)) AND ((Active Assisted) OR (Resistive) OR (Assist-As-Needed) OR (High Assistance) OR (Low Assistance) OR (Active Assistance) OR (Passive Assistance)) AND ((Upper Limb) OR (Upper Extremity) OR (Hand) OR (Arm) OR (Shoulder-Elbow) OR (Wrist) OR (Finger)) AND ((Rehabilitation) OR (Training) OR (Motor Training) OR (Therapy) OR (Recovery) OR (Functional Recovery)) AND (Control Group) NOT ((Review) OR (Animal) OR (Hip) OR (Lower Limb) OR (Leg) OR (Knee) OR (Foot) OR (Gait) OR (Walking) OR (Surgery) OR (Laparoscopy) OR (Cancer))

4. ((Active Assisted) OR (Resistive) OR (Assist-As-Needed) OR (High Assistance) OR (Low Assistance) OR (Active) OR (Passive)) AND ((Robot) OR (Robotic device) OR (Robot-assisted) OR (Robotic assistance) OR (Robotic training) OR (Robot-mediated) OR (Robot-based) OR (Robotic Rehabilitation) OR (End-effector) OR (Exoskeleton)) AND ((Upper Limb) OR (Upper Extremity) OR (Hand) OR (Arm) OR (Shoulder-Elbow) OR (Wrist) OR (Finger)) AND ((Rehabilitation) OR (Training) OR (Motor Training) OR (Therapy) OR (Recovery) OR (Functional Recovery)) AND (Control Group) NOT ((Review) OR (Animal) OR (Hip) OR (Lower Limb) OR (Leg) OR (Knee) OR (Foot) OR (Gait) OR (Walking) OR (Surgery) OR (Laparoscopy) OR (Cancer))

b) For advanced search in Scopus-

1. TITLE-ABS-KEY (("robo*" OR exoskeleton OR orthoses OR end-effector) AND (rehabilitation OR therapy OR training OR recovery) AND "assis*" AND ("upper limb" OR "upper extremity" OR hand OR arm OR "shoulder-elbow" OR wrist OR finger) AND "control group" AND NOT (review OR animal OR "lower limb" OR leg OR knee OR foot OR gait OR surgery OR laparoscopy OR cancer OR "follow-up"))

2. TITLE-ABS-KEY((“robo*” OR “exoskeleton” OR “orthoses” OR “brain-computer interface” OR “brain-machine interface”) AND (“assis*” OR “resis*”) AND (“upper limb" OR “upper extremity" OR “upper arm” OR hand OR arm OR “shoulder-elbow" OR wrist OR finger) AND (rehabilitation OR training OR “motor training" OR therapy OR recovery OR “functional recovery") AND (“control group”) AND NOT (review OR animal OR hip OR “lower limb" OR leg OR knee OR foot OR gait OR walking OR surgery OR laparoscopy OR cancer))

c) <u>For both advanced and manual search in Google Scholar multiple Boolean combinations of the following keywords were used-</u>

Robot, robotic device, robot assisted, exoskeleton, orthoses, assist, assistance, assistive, active assistance, passive assistance, high assistance, low assistance, unassisted, no assistance, resist, resistance, resistive, upper limb, upper extremity, hand, arm, wrist, finger, rehabilitation, neurorehabilitation, training, therapy.

Section-II

Graphical summary of the information extracted from the 13 included studies (11 on stroke, 1 on multiple sclerosis and 1 on spinal cord injury)

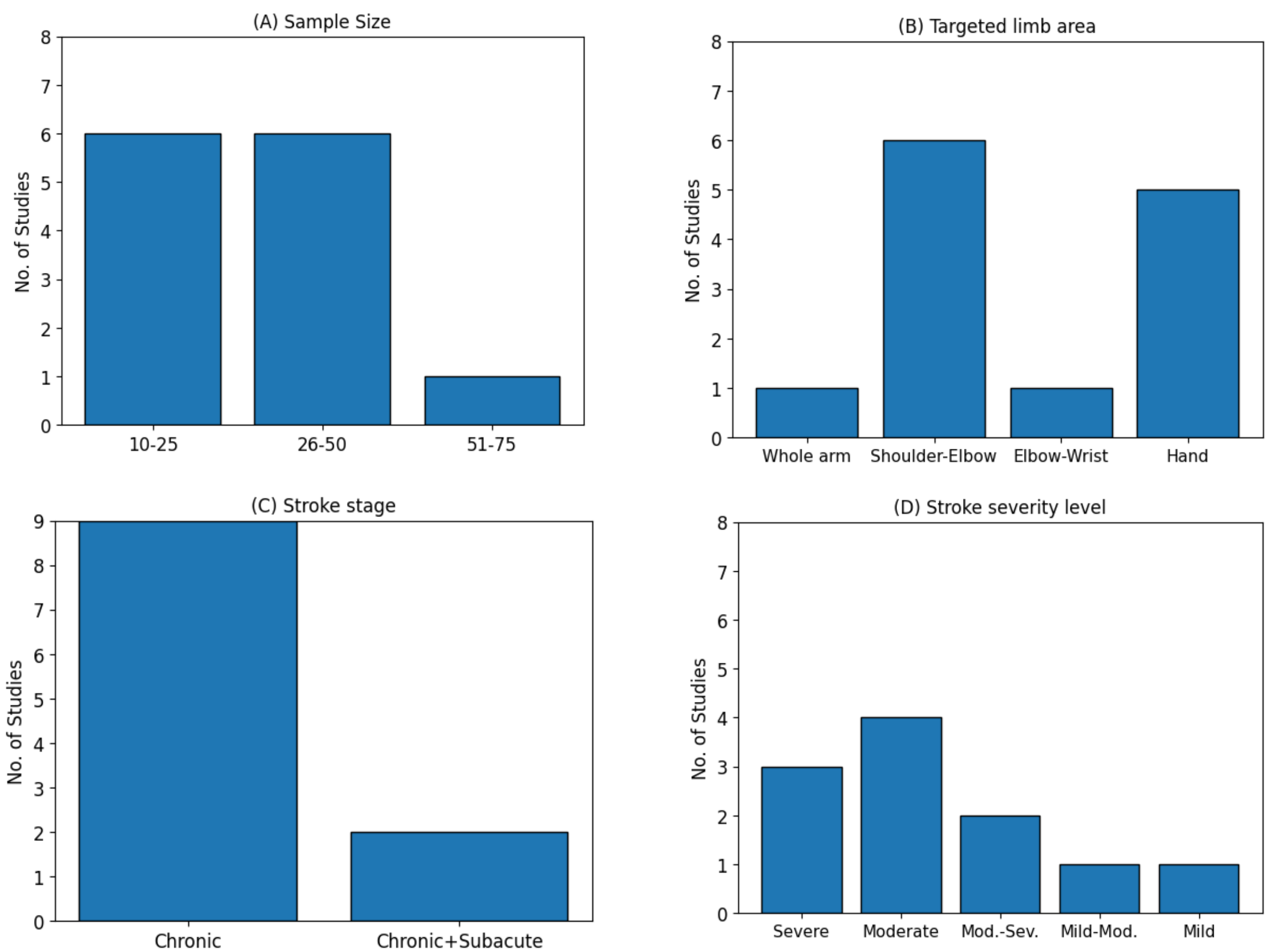

Figure 1: Histogram plots for (A) sample size, (B) targeted limb segment, (C) stroke stage and (D) stroke severity level across studies.

Section-III

A. For Meta-analysis

The following standard formulas were used to calculate the descriptive and inferential statistical parameters required for the meta-analysis.

Across studies, data for the primary outcome measure, the Fugl–Meyer Assessment (FMA) score, were extracted as mean ± standard deviation (SD). When change-score data were not reported in the required format, the mean change and corresponding SD were calculated from the reported pre- and post-intervention values using the methods described below[1]-

$$\text{Change in mean } (\Delta\mu) = \mu_{post} - \mu_{pre}$$

$$\text{Change in standard deviation } (SD_{\Delta}) = \sqrt{SD_{pre}^2 + SD_{post}^2 - 2 * r * SD_{pre} * SD_{post}}$$

Here, r is the correlation coefficient between the pre- and post-measurements; in none of the studies, the 'r' value was reported, so as per convention[1], it was considered as 0.7. The main analysis was also repeated for two different values of 'r' (0.5 and 0.9) to evaluate the robustness of the study outcomes to changes in the value of 'r' (results are provided in the subsequent section i.e., Section-IV).

For studies that reported the (a) standard error (SE), or, (b) 95% confidence interval (CI), or, (c) interquartile range instead of SD, the corresponding SD was computed as follows[2,3] –

$(a) SD = SE * \sqrt{no.of\ samples}$; or, $(b)\ SD = \sqrt{no.of\ samples} * \frac{(Upper\ limit - lower\ limit)}{D}$; or,

$$(c)\ SD = \frac{75\%\ quartile\ value - 25\%\ quartile\ value}{\eta}$$

Here, in equation *(b)*- the value of 'D' is considered 3.29, 3.92 and 5.15 for 90%, 95% and 99% confidence intervals respectively if the sample size is large; but if the sample size is small then the value of D is determined using t-statistics → 2*tinv (α, $n - 1$), where α is 0.05 for 95% confidence interval, and n is the no. of samples in the group. In equation *(c)*- η is a dividing factor which is a function of sample size, for sample size > 50, η value was considered as 1.35, and for sample size ≤ 50, η value varied[3] between 1.30-1.34.

Also, in a case, if median is reported instead of mean, then the mean was considered same as median for normally distributed sample, otherwise, the mean was computed as follows[3] –

$$Mean = \frac{75\%\ quartile\ value + median + 25\%\ quartile\ value}{3}$$

To obtain the summary effect of the combined outcomes of all/ a set of studies, following formulas[2,4,5] was used-

(i) $$SMD\ (\theta) = \frac{\mu_E - \mu_C}{\sigma_{pooled}} * j\ ;\ \sigma_{pooled} = \sqrt{\frac{(n_E - 1)\sigma_E^2 + (n_C - 1)\sigma_C^2}{n_E + n_C - 2}}\ ;\ j = 1 - (\frac{3}{4*(n_E + n_C - 2) - 1})$$

Where, $SMD(\theta)$is the standardized mean difference, μ_E is the mean change in pre-to-post intervention Fugl-Meyer Assessment (FMA) score change of experimental group, μ_c is the mean change in pre-to-post FMA score of control group; denominator is the pooled standard deviation, n_E is the no. of samples in experimental group, n_C is the no. of samples in control group, j is the Hedges' g correction factor for reducing the potential impact of small-sample bias.

(ii) $$SE_\theta = \sqrt{\frac{n_E + n_C}{n_E n_C} + \frac{\theta^2}{2(n_E + n_C - 3.94)}}\ ;\ CI_\theta = \theta \pm (1.96 * SE_\theta)$$

Here, SE_θ and CI_θ represent the standard error and confidence interval (95%) of SMD respectively

(iii) $$W_{i(FE)} = \frac{1}{SE_\theta^2};\ W_{Ni\ (FE)} = \frac{100 * W_{i(FE)}}{\sum_1^n W_{i(FE)}}$$

Here, $W_{i(FE)}$ and $W_{Ni\ (FE)}$ are the weights and normalized weights of the studies for fixed-effect (FE) analysis (inverse-variance method has been used for weight calculation)

(iv) Summary effect-size (SMD) of fixed-effect analysis: $\theta_{FE} = \frac{\sum_1^n (W_i * \theta_i)}{\sum_1^n W_i}$

Here, θ_i and $W_{i(FE)}$are the SMD and weight of ith study respectively; n is the total no. of studies

(v) Heterogeneity factors:

$$Q = \sum_{i=1}^{n} W_{i(FE)}\ (\theta_i - \theta_{FE})^2;\ \tau^2 = \max\left(0, \frac{Q - df}{\sum_1^n W_{i(FE)} - \frac{\sum_1^n W_{i(FE)}^{\ 2}}{\sum_1^n W_{i(FE)}}}\right);$$

$$I^2 = \max\left(0, \frac{(Q - df)}{Q}\right) * 100\%$$

Here, Cochrane Q quantifies the weighted dispersion of individual study effect estimates around the pooled effect and tests whether this variability exceeds that expected from sampling error alone; τ^2 is the estimated absolute between-study variance in the true effect sizes; I^2 estimates the

proportion of observed variability attributable to true heterogeneity rather than sampling error; and df is the degrees of freedom = total no. of studies *(n)* - 1

(vi) $$W_{i\,(RE)} = \frac{1}{SE_{\theta}^{2} + \tau^{2}};\; W_{Ni(RE)} = \frac{100 * W_{i(RE)}}{\sum_{1}^{n} W_{i(RE)}}$$

Here, $W_{i(RE)}$ and $W_{Ni\,(RE)}$ are the weights and normalized weights of the studies respectively for random-effect (RE) analysis

(vii) Summary effect-size (SMD) of random-effect analysis: $\theta_{RE} = \frac{\sum_{1}^{n}(W_{i(RE)} * \theta_{i})}{\sum_{1}^{n} W_{i(RE)}}$

Statistical significance of the results was obtained using the Z-score[4] for summary effects and Chi-square statistics for heterogeneity analysis. The respective formulas are as follows –

(viii) $$Zscore = \frac{summary\ SMD}{SE\ of\ summary\ SMD};\; SE\ of\ summary\ SMD = \frac{1}{\sqrt{\sum_{i=1}^{n} W_{i}}}$$

(ix) $$p\ value\ (two\ tailed\ test) = 2 * [1 - \emptyset(|Zscore|)]$$

where, W_i is the weight of the *i^th^* study, n = total number of studies, and $\emptyset$ is the standard normal cumulative distribution function

(x) $$p\ value\ (one\ tailed\ test) for\ heterogeneity = 1 - F_{\chi^{2}}(Q; df)$$

where, F_{χ^2} is the Chi-square distribution function, Q is the Cochrane-Q value – a measure of weighted dispersion of individual study effect estimates around the pooled effect, and df is the degrees of freedom = total no. of studies *(n)* – 1.

B. For publication bias estimation:

A scatter plot was obtained with effect size (as x value) and corresponding standard error (as y value) of individual studies. The summary effect was represented as the pooled random-effects of all the studies. The following formulas[6, 7] are used for linear regression (Egger's test) between precision (the inverse of standard error) and the standardized effect size, followed by intercept and p value computation.

$$\frac{\theta_{i}}{SE_{\theta_{i}}} = \beta_{0} + \beta_{1} * {}^{1}/_{SE_{\theta_{i}}} + \epsilon_{i}$$

Where, θ_i is the study-specific effect size (standardized mean difference), SE_{θ_i} is its corresponding standard error, $\frac{\theta_i}{SE_{\theta_i}}$ is the standard normal deviate of the study, $1/_{SE_{\theta_i}}$ precision of the ith study, β_0, β_1 and ϵ_i are the intercept, slope and residual error of the linear regression fit respectively.

$$p\ value\ (two\ tailed\ test) = 2 * (1 - F_t(|t|; n-2))$$

Where, F_t is the cumulative distribution function of Student's t-statistics; $t = \frac{\beta_0}{SE_{\beta_0}}$; (n-2) is the degrees of freedom; n is the total no. of studies.

A statistically significant intercept is interpreted as evidence of funnel plot asymmetry, although such asymmetry may arise from causes other than publication bias.

C. For Meta-regression

Exploratory univariable meta-regression was performed to examine whether individual study-level characteristics could explain part of the between-study heterogeneity observed in the main random-effects meta-analysis[8]. Each potential moderator was evaluated separately. The random-effects meta-regression model was specified as-

$$y_i = \beta_0 + \beta_1 X_i + u_i + \epsilon_i$$

where y_i is the observed effect size for the ith study, X_i is the value of the moderator, β_0 is the intercept, and β_1 is the regression coefficient representing the association between the moderator and the study effect size. The term ϵ_i represents within-study sampling error, with variance $v_i = SE_i^2$, whereas u_i represents residual between-study heterogeneity. The residual random effect was assumed to follow $u_i \approx N(0, \tau^2_{residual})$.

$\tau^2_{residual}$ denotes the residual between-study variance remaining after accounting for the moderator. Studies were weighted according to the random-effects inverse-variance weight (w_i) so that studies with greater precision contributed relatively more to the regression.

$$w_i = \frac{1}{v_i + \tau^2_{residual}}$$

The moderator coefficient was evaluated using $t = \frac{\beta}{SE(\beta)}$; where β is the regression coefficient and *SE* (β) is its standard error. A statistically significant coefficient was interpreted as evidence that the moderator was associated with variation in the study-level effect sizes.

The amount of between-study heterogeneity potentially accounted for by the moderator was estimated by comparing the between-study variance from the unadjusted random-effects model, $\tau^2_{unadjusted}$, with the residual between-study variance after inclusion of the moderator, $\tau^2_{residual}$:

$$R^2 = \frac{\tau^2_{unadjusted} - \tau^2_{residual}}{\tau^2_{unadjusted}} * 100\%$$

Here, $\tau^2_{unadjusted}$ represents the total estimated between-study variance before inclusion of the moderator, whereas $\tau^2_{residual}$ represents the heterogeneity remaining after adjustment for the moderator.

Residual heterogeneity was additionally assessed using the residual heterogeneity statistic Q_E:

$$Q_E = \sum_{i=1}^{k} w_i \left[y_i - (\beta_0 + \beta_1 X_i)\right]^2$$

A statistically significant Q_E indicated that heterogeneity remained among the study effects even after accounting for the moderator.

For categorical moderators, one category was treated as the reference category, and the remaining category was represented using an indicator variable. For continuous moderators, β_1 represented the expected change in effect size associated with a one-unit increase in the moderator.

Note: Multiple methods are available for performing random-effect meta-regression[9]. Among these, restricted maximum likelihood (REML) is one of the commonly used approaches. This method is generally preferred because it estimates the between-study variance using residual contrasts that are independent of the estimated fixed effects, yielding more accurate (less biased) variance estimates[9]. In finite samples, however, the REML-estimated $\tau^2_{residual}$ after inclusion of a moderator can exceed the $\tau^2_{unadjusted}$. This can occur because the two variance components are estimated separately and are subject to sampling variability, particularly when the number of studies and/or within-study sample sizes are small[9]. Consequently, the corresponding R^2, calculated from the proportional reduction in estimated heterogeneity, may be negative[9]. Such negative values are conventionally truncated to zero, indicating that the fitted moderator did not account for a detectable proportion of the between-study heterogeneity[9].

In the present study, REML was used. If the estimated R^2 was negative, it was truncated to 0. Given the small number of studies available, all meta-regression analyses were considered exploratory. Accordingly, non-significant moderator effects were interpreted as indicating insufficient evidence that the moderator explained between-study variation, rather than as evidence for the absence of a moderator effect.

The datasets generated and the python code developed for the analyses of this study can be accessed by the following link.
https://drive.google.com/drive/folders/1qi6-8ALz5OTooYSGrjuqGkR1SBK9RPXN?usp=sharing

The risk of bias (ROB) analysis document can be accessed by the link below.

https://docs.google.com/spreadsheets/d/1B243YBewx4PE0C5kHSxrFbsa_94shk28/edit?usp=sharing&ouid=114947611356048794179&rtpof=true&sd=true

Note: The ROB analysis was performed by answering the signaling questions using the standard Cochrane protocol[10]

Section-IV

A. Result for publication bias analysis

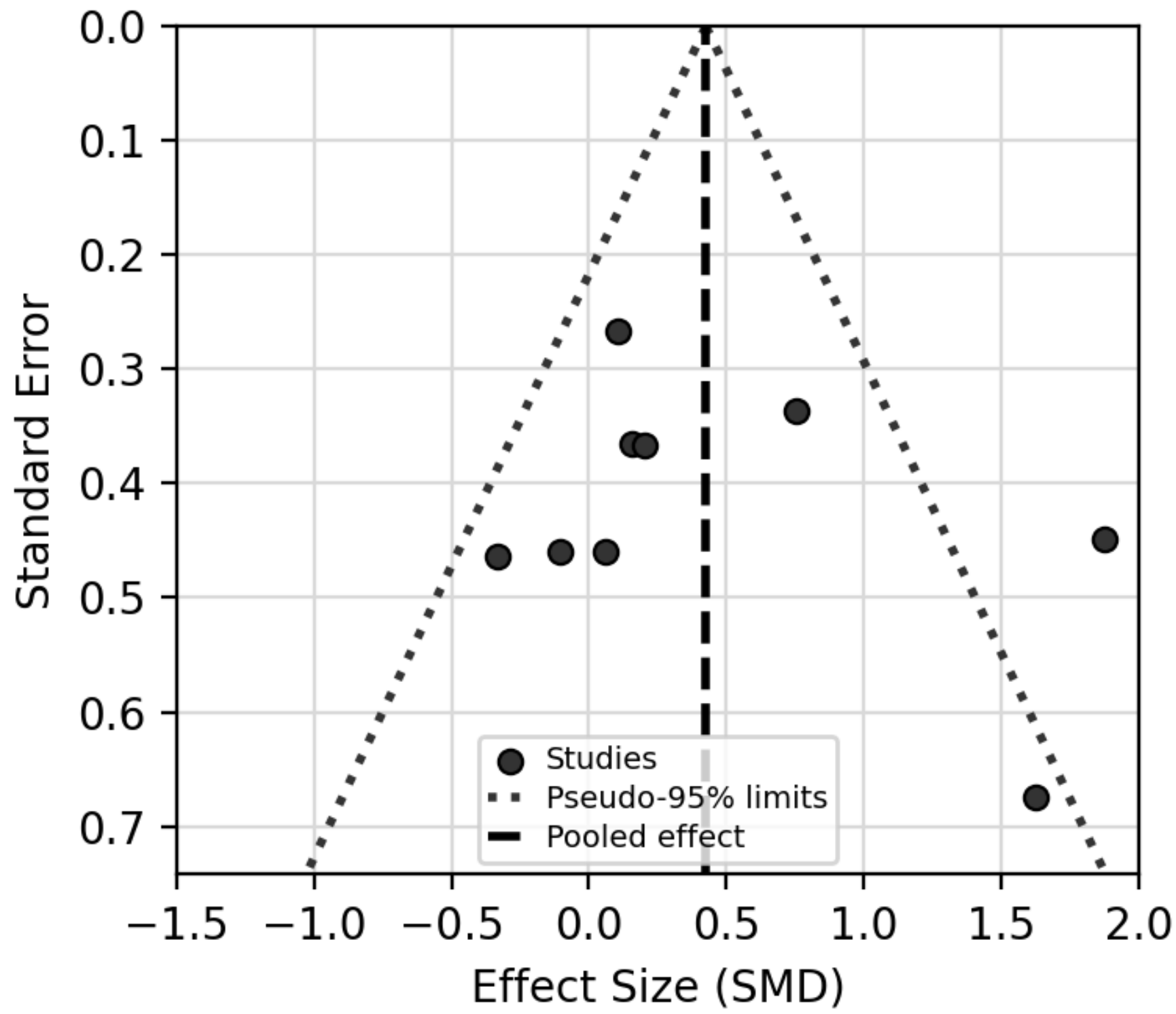

Figure 2: Assessment of funnel plot asymmetry as an indication of potential publication bias among the nine studies included in the main meta-analysis. Individual study effect sizes are plotted against their corresponding standard errors. The black dotted vertical line represents the pooled effect estimate, while the grey dotted lines indicate the pseudo-95% confidence limits around the pooled effect.

Observations: Visually, mild asymmetry can be seen in funnel-plot representation. The pooled effect size was 0.425 and Egger's regression yielded an intercept of 2.124 with SE: 2.317 and 95% CI: −3.35 to 7.60. Eight of nine studies lay within the pseudo-95% funnel limits. Overall, there was no statistically significant (p = 0.389) evidence of funnel-plot asymmetry or small-study effect, although publication bias cannot be ruled out given the small number of included studies.

B. Results for sensitivity analysis-

i) Meta-analysis results (9 studies (df =8); sample size 272) when correlation coefficient (r) between the pre and post measurements was considered as 0.5, 0.7 and 0.9

Main Analysis	FE-SMD with 95% CI	p value -FE	RE-SMD with 95% CI	p value -RE	Q	τ^2	I^2 (%)	p value -Q
**For r = 0.5**	0.351 [0.098, 0.604]	0.006	0.386 [0.006, 0.766]	0.047	16.79	0.170	52.36	0.0323
**For r = 0.7**	0.371 [0.116, 0.626]	0.004	0.425 [-0.007, 0.858]	0.053	21.40	0.263	62.62	0.006
**For r = 0.9**	0.391 [0.131, 0.65]	0.003	0.519 [-0.04, 1.078]	0.068	34.42	0.54	76.76	0.000034

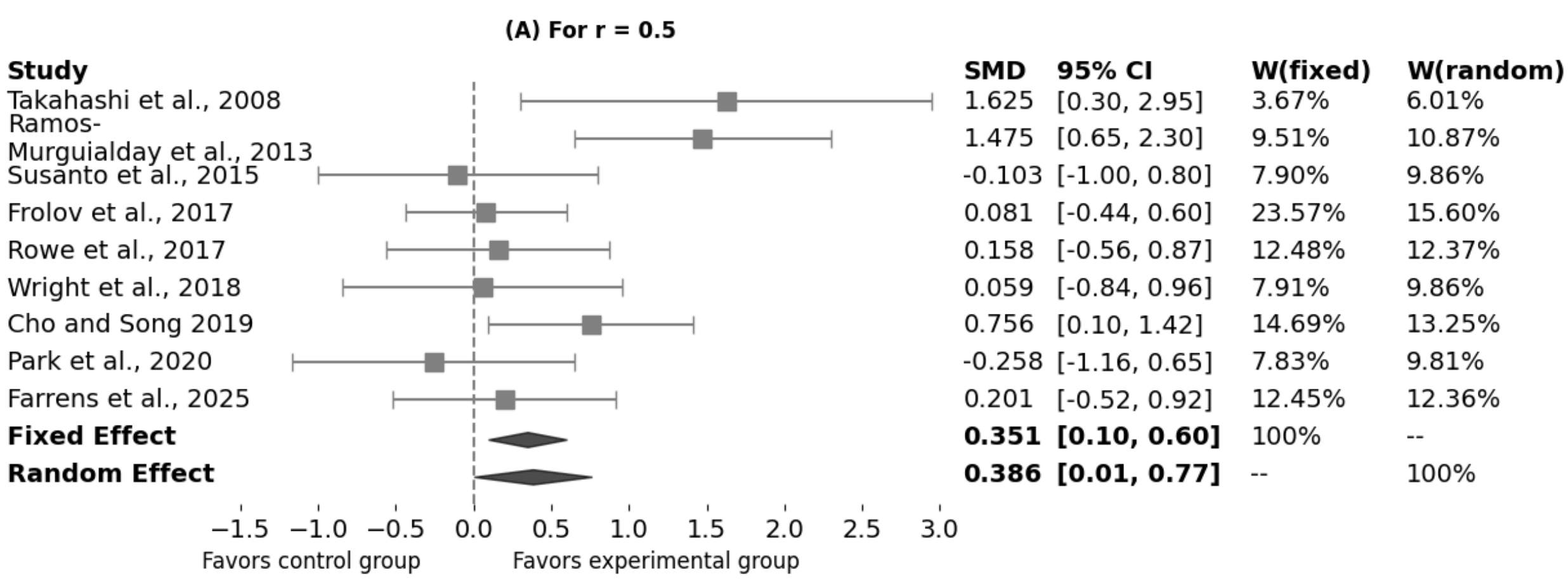

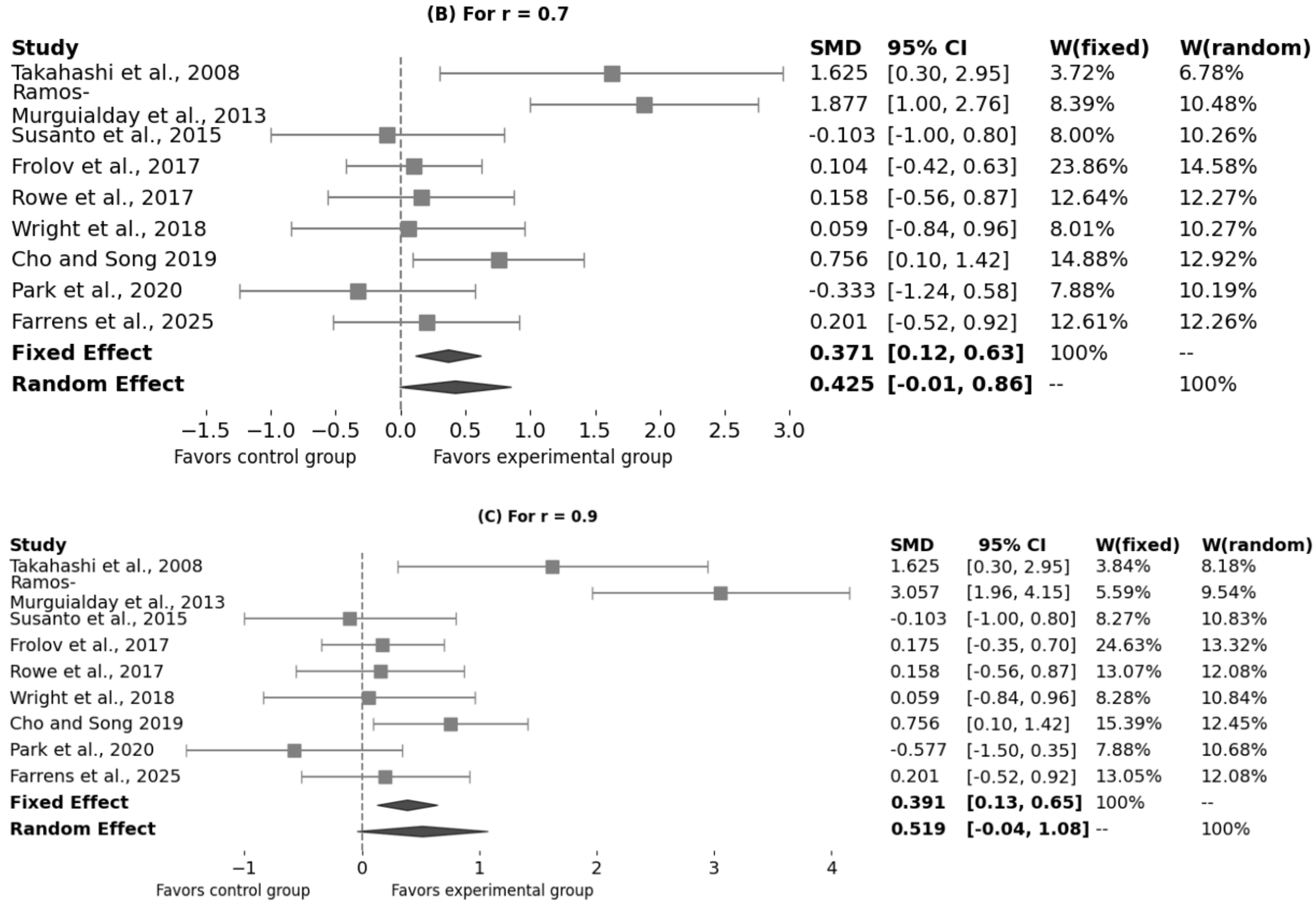

Figure 3: Forest plots for the robotic therapy meta-analysis on FMA scores when (A) r = 0.5, (B) r = 0.7, and (C) r = 0.9. For each study, the standardized mean difference (SMD) with its 95% confidence Interval (CI; rounded off to 2nd decimal place), and weights (W) for fixed-effect and random-effect models are given

Observations: Both fixed-effect and random-effect summary outcomes increase as 'r' increases, suggesting stronger observed effects when the correlation between pre- and post-measurements is higher**.** Higher correlation coefficients (r) result in larger effect sizes but introduce greater heterogeneity. The analysis with r = 0.9 yielded the largest effect-size estimates and the greatest heterogeneity, whereas r = 0.5 produced the most conservative estimates and lower heterogeneity. However, this comparative analysis indicates that the direction of the obtained summary effect is stable for low to high pre-post measurement correlation.

ii) Main meta-analysis report (10 studies; sample size - 286) that includes an additional study (Pila et al., 2021)[11] which apparently qualified the eligibility criteria but ultimately got excluded from core synthesis because of the ambiguity in its source reference, randomization and blinding procedure.

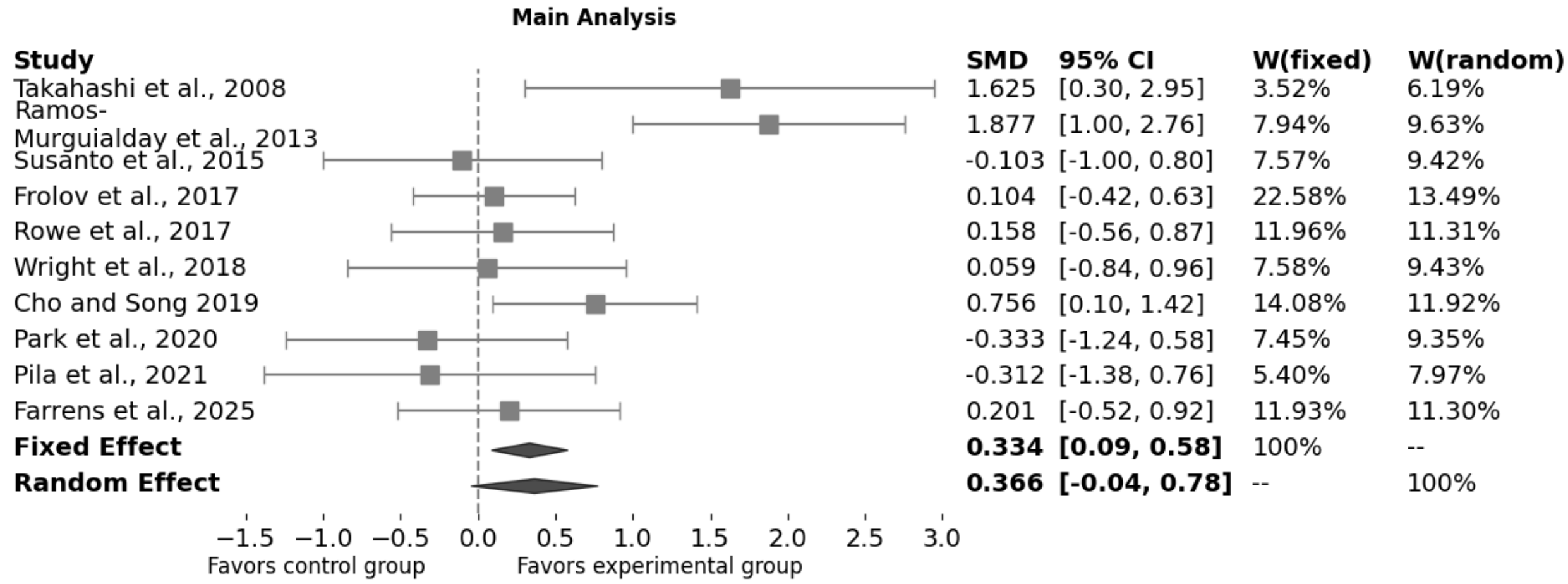

Figure 4: Forest plots for the robotic therapy meta-analysis on FMA scores of 10 studies. For each study, the standardized mean difference (SMD) with its 95% confidence Interval (CI; rounded off to 2nd decimal place), and weights (W) for fixed-effect and random-effect models are given

Observations: The obtained summary effect estimates were positive under both the fixed-effect model (SMD = 0.334, 95% CI: 0.086, 0.582) and the random-effects model (SMD = 0.366, 95% CI:-0.045, 0.777), favouring robotic assistance for upper-limb impairment recovery irrespective of disease stage, assistance level, or targeted limb segment. This indicates that the direction of the main effect remained stable following inclusion of the additional study. Under the fixed-effect model, inclusion of the additional study resulted in only a modest change in the summary effect estimate, from 0.371 to 0.334. Under the random-effects model, however, the pooled effect showed a comparatively larger reduction, from 0.425 to 0.366. Substantial between-study heterogeneity remained (I^2 = 60.68%, p = 0.0064), indicating considerable variability in effect estimates across studies. Thus, although the overall direction of effect remained favourable to robotic assistance, the magnitude and precision of the pooled estimate were influenced by the inclusion of the additional study.

iii) Influence analysis of the two large-effect studies- Takahashi et al.(2008) and Ramos-Murguialday et al. (2013). These two studies were removed and the main meta-analysis on remaining seven core studies (with N = 229) was repeated to observe the influence of these two studies on the pooled effect size, its directness, imprecision and heterogeneity.

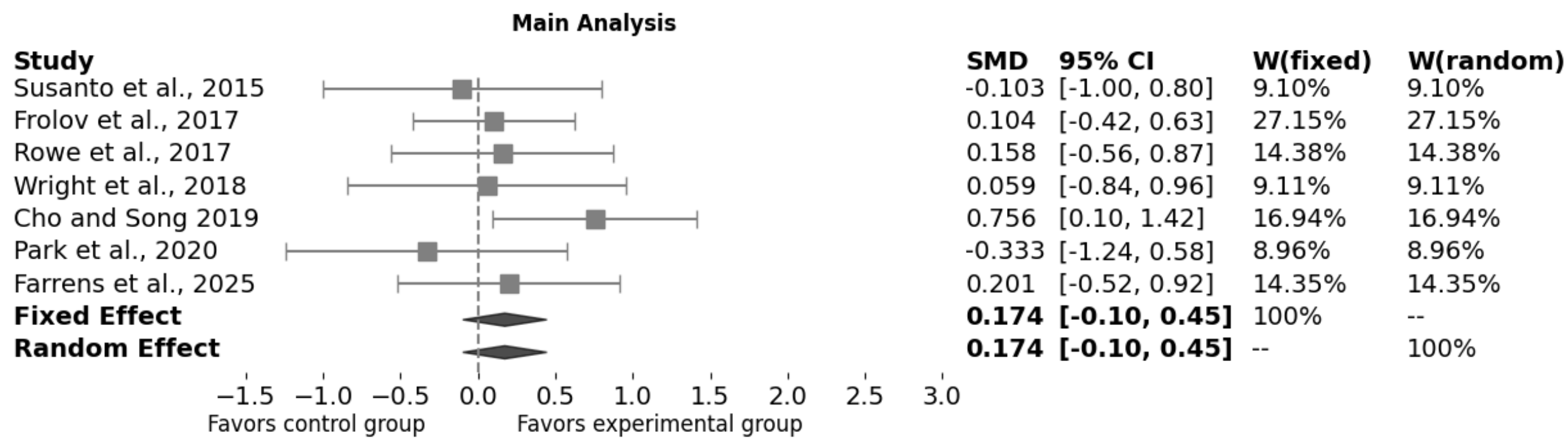

Figure 5: Forest plots for the robotic therapy meta-analysis on FMA scores of 7 studies after removal of two small-sample, large-effect studies. For each study, the standardized mean difference (SMD) with its 95% confidence Interval (CI; rounded off to 2nd decimal place), and weights (W) for fixed-effect and random-effect models are given.

Observations: Influence analysis showed that the pooled effect was substantially influenced by the two small-sample studies reporting large positive effects (Takahashi et al., 2008 and Ramos-Murguialday et al., 2013). In the primary analysis including all nine studies, the random-effects estimate favoured robotic assistance (SMD = 0.425; 95% CI: −0.007 to 0.858; p = 0.053), with substantial between-study heterogeneity. After excluding these two studies, the pooled effect decreased to SMD = 0.174; 95% CI:−0.098 to 0.446; p = 0.209, with I^2 = 0 %, Q = 4.677; p = 0.585. Although the direction of the pooled effect remained positive, its magnitude was markedly attenuated and the confidence interval continued to include the null. The concomitant reduction in the pooled effect and heterogeneity indicates that these two studies contributed substantially both to the magnitude of the overall estimate and to between-study variability. Accordingly, the apparent benefit of robotic assistance in the primary analysis should be interpreted cautiously, as it is partly driven by two small studies with comparatively large treatment effects. Also, the non-zero Q value indicates that some dispersion among the observed study effects remains, but because it is not statistically significant and I^2 = 0 %, this dispersion is consistent with that expected from sampling variability alone rather than providing evidence of residual between-study heterogeneity. However, the I^2 = 0 % in this case does not affirm that true heterogeneity is absent. Especially with a small number of studies, the analysis may simply lack power to detect it.

C. Results for meta-regression

Observations: Meta-regression analyses were conducted to investigate whether disease stage, limb segment, disease severity, and intervention dosage could explain between-study variation in effect sizes. Disease stage was not a significant moderator of treatment effect (p = 0.239; for df = 7) and explained only 7.3% (R^2) of the estimated between-study heterogeneity; the residual heterogeneity (Q_E = 17. 529; p = 0.014) was significant. Although a larger pooled effect was observed among studies involving participants with chronic disease compared with studies with mixed disease stages, the uncertainty surrounding the category estimates was substantial and the between-category difference was not statistically significant.

Limb segment was the only moderator to demonstrate a statistically significant association with effect size (p = 0.041; for df = 6), with non-significant residual heterogeneity observed among the studies (Q_E = 9.004; p = 0.173). The estimated intervention effect was greatest for studies involving the whole arm, followed by the shoulder–elbow and hand categories. Limb segment accounted for approximately R^2 = 95.0% of the estimated between-study heterogeneity, suggesting that the anatomical region targeted by the intervention may contribute to differences in treatment response. This result should, however, be interpreted cautiously because some limb-area categories contained very few studies, especially, the whole-arm category included only one study, resulting in imprecise category estimates and increasing the potential influence of individual studies.

Disease severity was not significantly associated with effect size (p = 0.727). Although differences in point estimates were observed across severe, moderate, mild, and mixed severity categories, confidence intervals were wide and substantially overlapping. The negative R^2 (-60.4% ≈ 0%) with significant residual heterogeneity (Q_E = 17.76; p = 0.003; for df = 5) indicated that including disease severity in the meta-regression did not explain the estimated between-study heterogeneity.

Similarly, intervention dosage showed no evidence of a linear association with effect size. The estimated regression coefficient (β_1) was close to zero (0.0044; p = 0.949), indicating that increasing dosage was not associated with a meaningful change in outcome measure. The negative R^2 (-26.0% ≈ 0%) with significant residual heterogeneity (Q_E = 21.393; p = 0.003; for df = 7) further indicated that dosage did not account for the between-study heterogeneity.

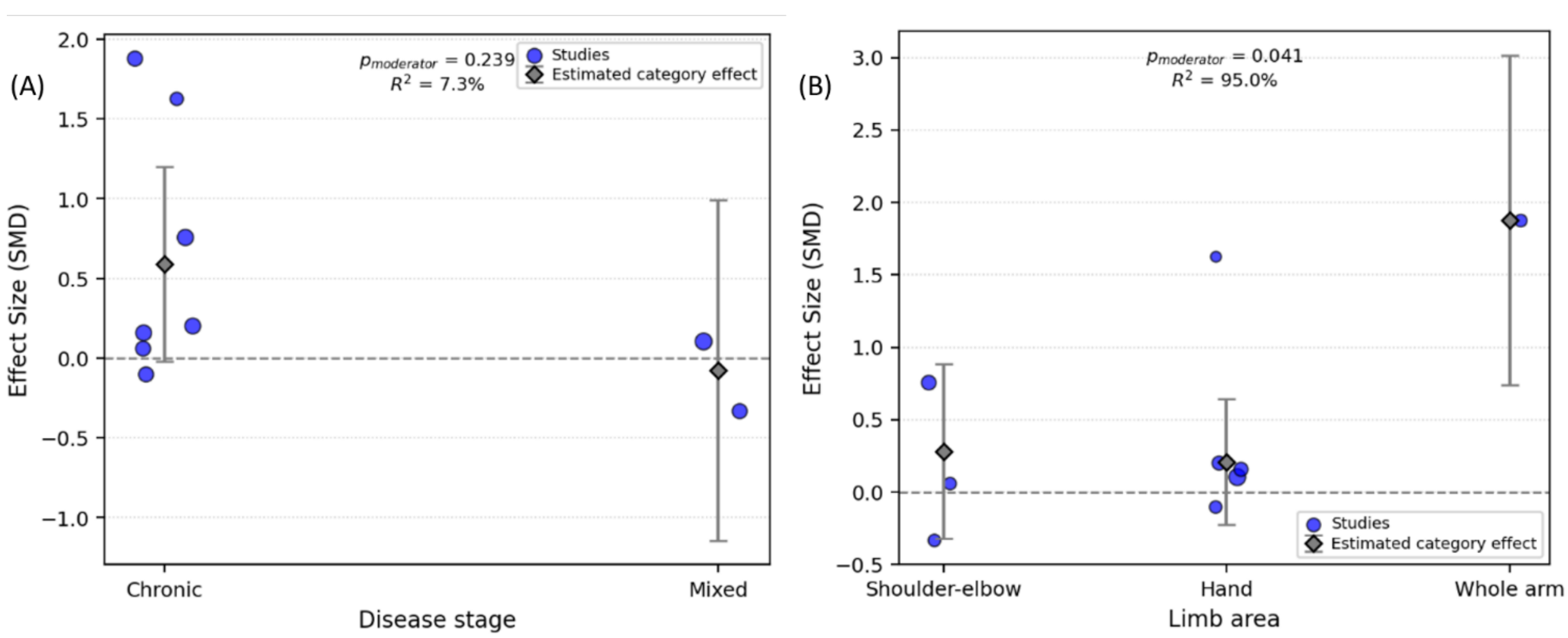

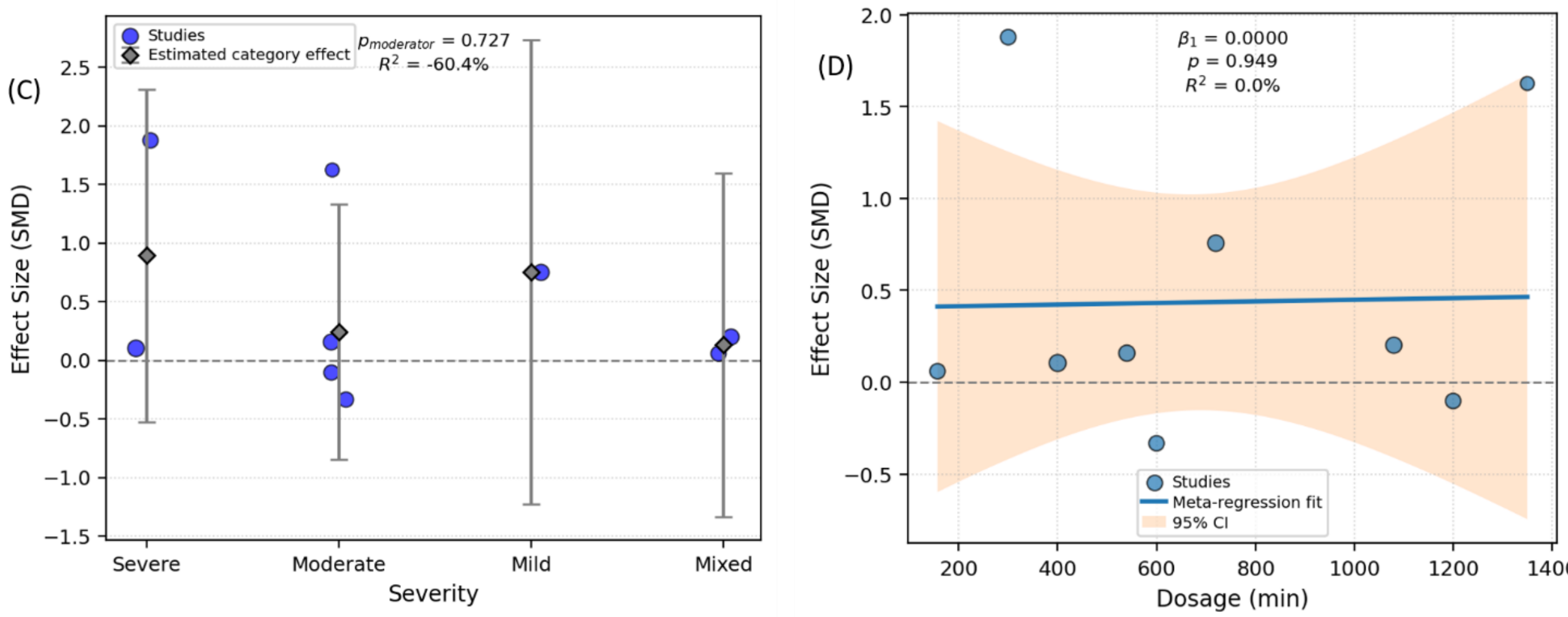

Figure 6: Meta-regression analyses of potential moderators of intervention effect. Meta-regression plots showing the association between standardized mean difference (SMD) and (A) disease stage, (B) limb segment, (C) disease severity, and (D) intervention dosage. For categorical moderators (A–C), circles represent individual study effect sizes, with circle size proportional to the study weight in the meta-regression, while diamonds represent the estimated category-specific effects with corresponding 95% confidence intervals. For the continuous moderator (D), circles represent individual study effect sizes, the solid line represents the fitted meta-regression relationship between dosage and SMD, and the shaded band represents the 95% confidence interval around the fitted mean effect. The moderator *p*-value assesses whether the moderator significantly explains variation in effect sizes, while (R^2) represents the proportion of between-study heterogeneity accounted for by the moderator.

References

1. Yagiz G, Akaras E, Kubis HP, Owen JA. The Effects of Resistance Training on Architecture and Volume of the Upper Extremity Muscles: A Systematic Review of Randomised Controlled Trials and Meta-Analyses. Applied Sciences (Switzerland). 2022;12(3). doi:10.3390/app12031593.
2. Higgins JPT, Li T, Deeks JJ, editors. Chapter 6: Choosing effect measures and computing estimates of effect. In: Higgins JPT, Thomas J, Chandler J, Cumpston M, Li T, Page MJ, et al., editors. Cochrane Handbook for Systematic Reviews of Interventions. Version 6.5. Cochrane; 2024.
3. Wan X, Wang W, Liu J, Tong T. Estimating the Sample Mean and Standard Deviation from the Sample Size, Median, Range and/or Interquartile Range. Vol 14.; 2014. http://www.biomedcentral.com/1471-2288/14/135.
4. Deeks JJ, Higgins JPT, Altman DG, McKenzie JE, Veroniki AA. Chapter 10: Analysing data and undertaking meta-analyses. In: Higgins JPT, Thomas J, Chandler J, Cumpston M, Li T, Page MJ, Welch VA, editors. Cochrane Handbook for Systematic Reviews of Interventions. Version 6.5. Cochrane; 2024.
5. Yıldırım Y, Şahin MG. How do different weighting methods affect the overall effect size in meta-analysis? An example of science attitude in Türkiye sample. Int J Psychol Educ Stud. 2023;10(3):744-757. doi:10.52380/ijpes.2023.10.3.1049.
6. Lin L, Chu H. Quantifying publication bias in meta-analysis. Biometrics. 2018;74(3):785–794. doi:10.1111/biom.12817.
7. Egger M, Davey Smith G, Schneider M, Minder C. Bias in meta-analysis detected by a simple, graphical test. BMJ. 1997; 315:629–634. doi: 10.1136/bmj.315.7109.629.

8. Thompson SG, Higgins JPT. How should meta-regression analyses be undertaken and interpreted? Stat Med. 2002; 21:1559–1573. doi: 10.1002/sim.1187.
9. López-López JA, Marín-Martínez F, Sánchez-Meca J, Van den Noortgate W, Viechtbauer W. Estimation of the predictive power of the model in mixed-effects meta-regression: a simulation study. Br J Math Stat Psychol. 2014; 67:30-48. doi:10.1111/bmsp.12002.
10. Higgins JPT, Savović J, Page MJ, Elbers RG, Sterne JAC. Chapter 8: Assessing risk of bias in a randomized trial. In: Higgins JPT, Thomas J, Chandler J, Cumpston M, Li T, Page MJ, Welch VA, eds. Cochrane Handbook for Systematic Reviews of Interventions. Version 6.5. Cochrane; 2024. Accessed April 11, 2025. https://www.training.cochrane.org/handbook.
11. Pila O, Koeppel T, Duret C, Jamin P. Comparison of active-assisted and active-unassisted robot-mediated upper limb therapy in subacute stroke. Restor Neurol Neurosci. 2021;39(1):1-11. doi:10.3233/RNN-201010.

Section-V

Summary of the details of the training provided by the studies chosen for the systematic review-

Study	Robotic Assistance	Movement target details	Movement characteristics emphasised	Movement details	Training feedback	Comments
Takahashi et al., 2008	**EG:** Full active assistance (A-A) throughout (15 days or 15 sessions)<br>Hard stop of the robotic assistance depended on patient's passive ROM<br><br>**CG:** 7.5 days of training (half of the sessions) - active non-assist mode (ANA-A), but connected to the device, another 7.5 days of training - active assistance mode (A-A)	Grasp and release movement (11-15s long each).<br>For A-A: subjects were given 1-3s time to attempt to open or close fingers, after that assistance provided by the robot.<br>For ANA-A: subjects were given 3-5s time to attempt to open or close fingers<br><br>No information about the grasp object given	Game rule emphasized control of movement range, speed and timing. Accordingly, game difficulty levels were adjusted to avoid floor and ceiling effect<br><br>Also, task-specific motor cortex reorganization observed in fMRI analysis	9 cycles of 10 repetition in first half of each day's session, then15 min break, followed by second half of the session, wherein, subjects played VR based 9 games focusing grasp and release movement; A-A mode training with robotic assistance and ANA-A without assistance	Visual feedback given through LCD monitor, Audio instructions were given for grasp and release – 'get ready', 'close', 'open', 'rest'.	Study can be replicated from the details given about the intervention.<br><br>However, grasp strength and grasping object information are missing<br><br>Blinding information not mentioned clearly<br><br>In inclusion criteria, stroke onset time mentioned as at least > 3 months; mean and range of onset time are mentioned incorrect; it is confusing that included subjects all were chronic or mixed of subacute and chronic

Ellis et al. 2009	**EG:** Progressive shoulder abduction loading<br>**CG:** Full shoulder abduction support<br>Robotic support but no assistance	Discrete planar reaching movements. One target each in 5 different reaching directions.<br><br>No clear information about the location of the targets	Reach extent was emphasized; getting as close to the target is better.	3 sets of 10 repetitions for each movement direction<br>No mention of the typical duration of each session.<br>30 second rests between repetitions, and 1 minute rest between sets.	Subjects received visual feedback from a computer monitor about trajectory of the actual movement. Occasional/random feedback of movement performance (not clear what this feedback was about) was also given. No mentioned on the feedback schedule.	Details about target locations is missing. The robot does not provide active assistance for training; however, provided arm-weight support which aided the patients while performing the task.<br><br>Seeing change in FMA would have been interesting.
Huang and Patton 2013	3 sessions of training<br><br>**EG:** Two equal sub groups (cross over design)- Null force (no external force -N), negative viscous force (velocity dependent destabilizing force -V), and negative viscous force with inertia (Combined -C)<br><br>Condition randomization – NVC or NCV<br><br>**CG:** Null force field (NNN)	**Training phase-** any movement of own choice using a variety of directions, speed and positions within a rectangular workspace (exploratory movement for 25 min)<br><br>**Evaluation phase-**four complete counter-clockwise revolutions around a target (circular tracking movements)	Speed, accuracy and smoothness of movement	2 hours 15 min session duration. Each session included several alternating training phases (16) and evaluation trials (160). Experimental protocol not clear. No clear information about movement repetitions	Real-time feedback of the handle position, visual reference cues, and experiment instructions were presented on a horizontal surface overlying the planar workspace of the arm.	Study can be replicated from the details given about the intervention.<br><br>Blinding information not mentioned clearly<br><br>Groups were not balanced in terms of the severity level of the patients as the baseline FMA score for all the patients were not available/ measured
Ramos-Murguialday et al., 2013	**EG:** robotic assistance contingent on movement intension (concurrent movement of orthoses)<br><br>**CG:** robotic assistance non-contingent on movement intension (random movement of orthoses)	The patients were instructed to move their arm and try to reach forward (even if the robotic arm orthosis does not follow their intension), then grasp, and bring an imaginary apple to their lap, thus involving finger extension during the reach and grasp movement. Each trail was of 5s.<br><br>After 8th session, the BMI training was switched from arm to hand.	Not clear what aspects of the movement were emphasized	Approximately 13 – 15 min session duration (estimated by the reader; no distinct information provided by the authors).	No mention about feedback for task result or task performance	How the concurrent movements of the arm and hand orthoses took place that is not clear. Also, no information about reaching distance, grasp size.<br><br>Study may not be replicated with the information provided

Susanto et al., 2015	**EG:** robot (exoskeleton) assisted<br><br>**CG:** attached to the exoskeleton but unassisted	Three modes of training- hand grasp-open (4 min), three-finger pinch-open (8 min), two-finger pinch-open (8 min); 1-2 min rest after each block. In all cases, subjects were instructed to move a kitchen sponge on a horizontal plane along a predefined direction within the predefined training space	Not clear what aspects of the movement were emphasized (speed, accuracy, quality)<br><br>Improvement in number of repetitions was one of the outcome measures	1 hour session duration. Task related information is sufficiently provided	Verbal instructions and feedback were given; start and end points of movements were marked for subjects' ease of doing the task	The study can be replicated from the details given about the intervention.<br>The control group didn't get assistance from robot but got it from therapist.<br>The result may be confounded due to the therapist assistance; role of robotic assistance is obscured.<br>Single blinded study
Frolov et al., 2017	**EG:** Assistance dependent on motor imagery-based EEG activity<br>**CG:** Assistance non-dependent on motor imagery-based EEG activity; sham BCI	Subjects performed three mental tasks- motor relaxation, motor imagery of paretic hand opening, and motor imagery of non-paretic hand opening.<br>**BCI active-** the task-imagination specific brain activity drove the exoskeleton attached to paretic hand<br>**BCI passive-** actuation of the exoskeleton based on the cue (appeared arrow on the screen), not based on the brain activity	Not clear what aspects of the movement were emphasized (speed, accuracy, quality)	40 min session duration<br>Visual instructions on a computer screen were provided, wherein three coloured arrows indicated which mental task to be performed when.	The subjects received both visual and kinaesthetic feedback of the results of BCI decoding of their imagery attempts	The study can be replicated from the details given about the intervention.
Frullo et al., 2017	**EG:** Assist as needed- triggered assistance when movement stops<br><br>**CG:** Self-triggered control (partial passive assistance); full robotic assistance was provided when subject initiated movement crossed a threshold force towards intended direction	Subjects performed point to point reaching movement from centre position to peripheral target position (in the active ROM)	Movement velocity and number of repetitions	90 min session duration. Not clear how much time was spent moving and resting	Subjects received visual feedback from a computer screen (home position, target position, desired trajectory, direction to which force should be applied)	The study can be replicated from the details provided about the intervention<br><br>Blinding information not mentioned clearly

Rowe et al., 2017	**EG:** High assistance; (causing 80-85% success rate at hitting targets)<br>**CG:** Low assistance; (causing 50-55% success rate at hitting targets)<br>The amount of robotic assistance was varied between groups while number, amplitude and exerted effort of training movements were controlled.	Robot assisted the participants by applying guiding forces to direct their fingers along a physiological spatiotemporal trajectory, ensuring they reached the musical note at the target. However, these assistive forces were only activated when participants initiated the movement themselves, as detected by force sensors positioned between their fingers and the robot mechanisms, with a threshold set at 6 N.	Movement trajectory and force.<br>Movement trajectory intercepted the musical note at the target.	1 hour session, moving index and middle finger to the targets to play a musical game. Total of 1065 possible movements per session	Subjects received visual feedback from a computer screen about the trajectory, moment of crossing and the angular distance to the actual crossing	The study can be replicated from the details provided about the intervention given to the subjects.
Wright et al., 2018	**EG:** Deficit force-field robotic assistance<br><br>**CG:** Null force-field robotic assistance	**Training-** two blocks of eight, 2 min motor exploration trials (total 32 min) During exploratory movement task, a trial was ended after two cumulative minutes of movement with speed > 0.04m/s; 1-3 min intermittent rest period<br>**Performance assessment**- 30 trials goal directed reaching task; 6 trials circular movement task; 6 trials - exploratory movement task	Movement trajectory and velocity	Training duration in a session- 33 to 35 min including rest.<br>Task related information is adequately given	Real-time visual feedback of the robot's handle location<br>Additionally, randomness score for exploratory movement	Study can be replicated with the information provided about the intervention<br><br>Blinding information not mentioned clearly
Cho et. al 2019	**EG:** Assist-as-needed mode - triggered assistance (maximum 4N assistive force); when movement stops for 2 sec, assistance is ON<br><br>**CG:** Guidance mode- corrective and assistive forces (maximum 4N) were applied during reaching to a straight-line path. Assistance was always ON.	Discrete 3D reaching movements; 6 different targets in 6 directions at desk and chest level<br><br>No mention of where the targets were located with respect to the subject; no information about reaching distance for individual target<br><br>Not clear how target locations were scaled to subject's arm length	Not clear what aspects of the movement were emphasized (speed, accuracy, quality).	40 min session duration. It is not clear how much time was spent moving and resting. No mention of the number of movement repetitions	Subjects received visual feedback from a computer screen about the trajectory of the actual movement. Audio feedback on successful target reach.	The study cannot be replicated from the details provided about the intervention given to the subjects.<br><br>No clear mention about subjects' severity. Not clear- Does "subjects were all high functioning" imply they were mildly affected?
Solaro et al., 2020	**EG:** Haptic- robot assisted<br>**CG:** Sensorimotor- visual control- no force applied by the robot	Fast and accurate point to point (22.5 cm) reaching task in 6 different directions (0º, 60º, 120º, 180º, 240º, 360º)	Movement trajectory and reaching time	40 min session duration. 8 epochs in session; in each epoch- 4 movement repetitions for each direction (thus, total 24 movements).<br>No information about rest time	Subjects received visual feedback from a computer screen.<br>Auditory feedback was also provided after task completion (pleasant or unpleasant sounds depending	Although subjects of control and experimental group performed same task, the difficulty level was greater for experimental group<br><br>Study can be replicated from the

					on the score).	details provided about the intervention
Park et al., 2020	**EG:** Active robotic assistance with gravity compensation<br>**CG:** Unassisted; only mechanical support with gravity compensation by the robot; no actuation	Subjects were trained with a game-based virtual reality environment with a focus on proximal upper limb movement.	Not clear what aspects of the movement were emphasized (speed, accuracy, quality).	30 min session. No information about the games, movement type, direction, number of repetitions and rest period	Subjects received visual feedback from a computer screen.	The study can be replicated from the details provided about the intervention given to the subjects.<br><br>Blinding information not mentioned clearly
Farrens et al., 2025	**EG:** Active robotic assistance (physical guidance force)<br>**CG**: Virtual assistance (No physical guidance force; assistance through amplifying display movements and by adjusting gaming speed and accuracy requirement)	Subjects were trained with a game-based computer interface for finger movements. Two games (Rehab hero- music note hitting and finger pong) used for training flexion/extension of index and middle fingers and thumb circumduction	Movement timing and speed	Each session was of 2 hours; rest duration not reported. 80% success rate with ~1050 repetitions/ sessions were maintained	Subjects received visual feedback from a computer screen.<br><br>Real-time visualization of hand movement was occluded.	The study can be replicated from the details provided about the intervention given to the subjects.<br><br>Blinding information not mentioned clearly

Note: For all the included RCTs, participants in the experimental and the control groups actively practiced the same task. However, differences in voluntary effort may have occurred between the groups as a direct consequence of the assistance contrast being evaluated in each RCT.
